# Water-sediment regulation drives stage-specific microbial shifts and network complexity in the Yellow River reservoir-river continuum

**DOI:** 10.3389/fmicb.2025.1640934

**Published:** 2025-10-29

**Authors:** Yanmin Zhang, Bo Zhao, Zewei Gui, Man Zhang, Xiaofei Gao, Xulu Chang, Guokun Yang, Xiaolin Meng, Hongchen Jiang

**Affiliations:** ^1^College of Fisheries, Henan Normal University, Xinxiang, China; ^2^School of Life Sciences, Henan University, Kaifeng, China

**Keywords:** water-sediment regulation, microbial community, reservoir-river continuum, Yellow River, molecular ecological networks

## Abstract

High-turbidity rivers, exemplified by the Yellow River, face significant ecological risks due to anthropogenic water-sediment regulation (WSR), which disrupts sedimentary habitats and biogeochemical cycles. However, the stage-specific impacts of WSR on microbial community structure, network complexity, and biogeochemical functions in reservoir-river continua remain poorly understood. In this study, we investigated microbial responses across different WSR stages in the Xiaolangdi Dam reservoir-river continuum using an integrated approach, including 16S rRNA gene sequencing, molecular ecological network analysis (MENs), and hierarchical partitioning. The results showed that WSR induced transient but profound shifts in microbial communities. The sediment-regulation stage (Inter_WSR3) exerted the strongest disturbance, characterized by peak turbidity (77.80 NTU), nutrient fluxes (NO_3_^−^ = 3.10 mg/L), and sediment resuspension, which restructured surface sediment (SS) communities dominated by copiotrophic *Gammaproteobacteria* (35.69%) and *Bacteroidia* (14.82%). Microbial α-diversity transiently increased during WSR but recovered to baseline levels post-disturbance, masking β-diversity divergence driven by niche differentiation. Molecular ecological networks exhibited peak complexity (nodes = 1,318; modularity = 0.73) during Inter_WSR3 but failed to recover Post_WSR, reflecting weakened functional redundancy and ecosystem resilience. Hierarchical partitioning identified stage-specific drivers: chlorophyll a (Chla) dominated SS assembly during Inter_WSR3, while nitrate (NO₃^−^) and turbidity governed particle-attached (PA) and free-living (FL) communities. Light limitation and sediment-water interactions overrode dissolved oxygen and temperature as primary drivers in the Yellow River. These findings reveal that WSR disrupts microbial co-occurrence patterns and functional redundancy, with lasting consequences for ecosystem services. To reconcile sediment management with ecological sustainability, we advocate phased WSR implementation, targeted monitoring of FL/PA communities, and habitat restoration to enhance connectivity. This study advances the mechanistic understanding of high-turbidity river ecology and provides actionable insights for global river management.

## Introduction

The Yellow River, often lauded as the “cradle of Chinese civilization,” presents a study in biogeochemical complexity, owing to its status as one of the world’s most sediment-laden waterways, annually transporting in excess of 1.6 billion tons of suspended sediment (Chen et al., 2021). This characteristic high turbidity, a consequence of pronounced erosion within the Loess Plateau, imposes significant ecological constraints upon the riverine ecosystem, particularly amidst anthropogenic pressures such as dam construction and water-sediment regulation (WSR). Since the 1960s, the implementation of large-scale reservoirs along the river’s course, including the Xiaolangdi Dam (XLD), has profoundly altered the hydrological regime and biogeochemical cycles of the Yellow River (Shi et al., 2017). WSR, an operational strategy undertaken annually by the Yellow River Conservancy Commission (YRCC), involves the strategic release of sediment-rich water to mitigate downstream sedimentation and facilitate navigation (Zhao et al., 2022). However, this practice creates extreme hydrological and biogeochemical gradients. These include episodic nutrient flows, sediment suspensions, and light-emitting factors that may impair the integrity of microbial communities, which are key drivers of biogeochemical processes in rivers (Maavara et al., 2020; Wang et al., 2022).

Despite the pivotal role of microbial communities in mediating carbon, nitrogen, and phosphorus cycles, knowledge of their response to WSR within highly turbid river systems remains limited. Previous research has mainly focused on less turbid systems, such as the Yangtze River, where microbial communities and functions differ markedly from those in more turbid environments (Liu Q. et al., 2018; Wang et al., 2021b; She et al., 2022). For instance, chlorophyll a (Chla) and dissolved oxygen (DO) are established determinants of bacterial community assembly in relatively clear-water rivers (Green et al., 2012; Peiffer et al., 2021). In stark contrast, highly turbid systems such as the Yellow River are characterized by light limitation, elevated concentrations of suspended particulate matter, and pronounced sediment-water interactions, thus fostering distinct microbial assemblages adapted to these conditions (Xia et al., 2013; Pan et al., 2022a, 2022b). Recent investigations have highlighted the prevalence of phyla such as *Proteobacteria*, *Bacteroidetes*, and *Actinobacteria* within Yellow River sediments (Song et al., 2012; Xia et al., 2014), with particle-attached (PA) and free-living (FL) microbial communities demonstrating seasonal fluctuations contingent upon hydrological variability (Pan et al., 2022a, 2022b). Nevertheless, the precise mechanistic linkages between WSR-induced environmental perturbations and subsequent microbial community restructuring within reservoir-river continua remain elusive.

A critical knowledge gap exists concerning the differential responses of surface sediment (SS), PA, and FL microbial communities to WSR operations. Sediments function as hubs of microbial activity and nutrient cycling, while PA microorganisms contribute to particle aggregation and organic matter degradation (Crespo et al., 2013; Yeh and Fuhrman, 2022). Conversely, FL microorganisms are more directly influenced by hydrodynamic conditions and the availability of dissolved substrates (Palmer and Ruhi, 2019). The interdependent impacts of WSR on these three compartments and their respective contributions to biogeochemical cycling remain largely unexplored. Furthermore, while some studies have documented short-term shifts in microbial diversity in response to WSR (Song et al., 2022), the long-term resilience of microbial networks and their feedback mechanisms to sediment pulses remains poorly understood. Addressing these knowledge gaps is crucial to optimizing WSR strategies, thereby striking a balance between sediment management objectives and ecological sustainability within high-turbidity river systems.

We hypothesize that WSR precipitates stage-specific alterations in microbial community structure and network complexity across the SS, PA, and FL compartments within the Yellow River reservoir-river continuum. Furthermore, we posit that the sediment-regulation stage (Inter_WSR3) will exert the most pronounced disturbance, attributable to the pulsed discharge of sediment, thereby inducing transient increases in microbial richness and alterations in network topology. We additionally hypothesize that stage-specific physicochemical gradients [e.g., Chla, NO₃^−^, dissolved inorganic carbon (DIC)] will drive compositional divergence among SS, PA, and FL communities. This study aims to test these hypotheses using a spatiotemporal survey that encompasses different WSR stages in the Xiaolangdi Dam reservoir-river continuum. The investigation employed 16S rRNA gene sequencing, molecular ecological network analysis (MENs), and hierarchical partitioning to evaluate these dynamics. The objectives of this study are to (1) quantify the temporal dynamics of α-diversity, β-diversity, and microbial interactions in SS, PA, and FL compartments; (2) identify stage-specific environmental drivers shaping microbial community structure; and (3) elucidate the legacy effects of WSR on microbial network complexity and ecosystem functioning.

## Materials and methods

### Study area, field measurements, and sampling

**Figure 1:**
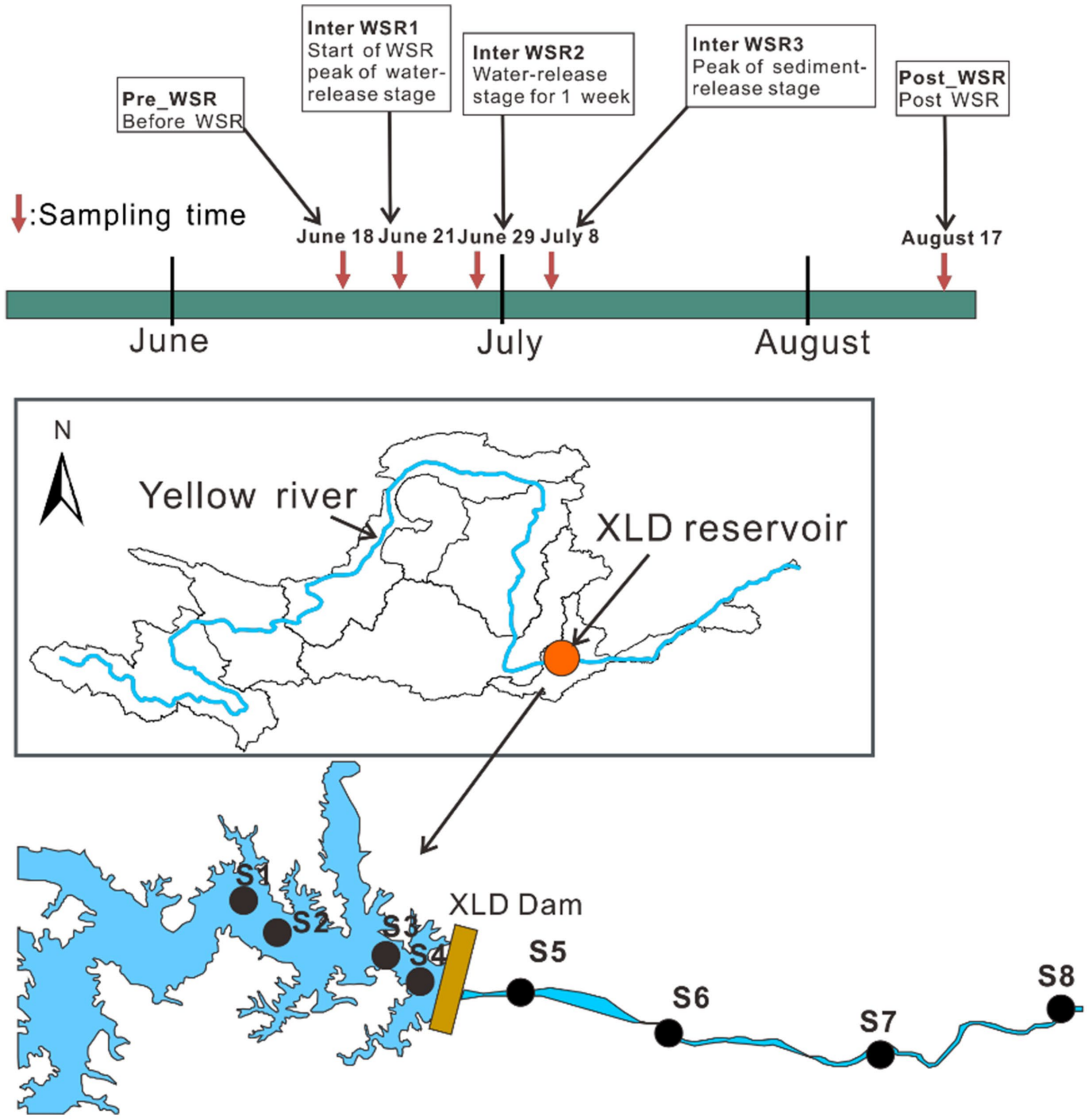
Setting of sampling times and sampling sites in this study. Five typical sampling stages of WSR (Pre_WSR, Inter_WSR1, Inter_WSR2, Inter_WSR3, and Post_WSR), from June to August, along with eight sampling sites along the reservoir-river continuum of the main stem of the Yellow River, were included in this study. S1–S4 represent sites within the XLD reservoir, while S5–S6 represent sites located downstream of the XLD reservoir.

To explore the response of the microbial community structure to the environmental factors within the reservoir-river continuum ecosystem of XLD during the WSR process, five representative periods from June to August in 2023 were selected as the sampling times, including Pre_WSR (June 18th, before WSR), Inter_WSR, which includes Inter_WSR1 (June 21st, the start of water regulation, the peak water-release stage of WSR), Inter_WSR2 (June 29th, when water regulation lasted 1 week), Inter_WSR3 (July 8th, the start of sediment regulation, the peak sediment-release stage of WSR), and Post_WSR (August 17th, post-WSR). Eight sampling sites (S1–S8) were selected, four of which were in the XLD reservoir and four of which were downstream of it. The final sampling site was located upstream from the confluence of the Yiluo River to minimize the influence of tributaries (Figure 1). No extreme weather was observed during the sampling period.

Water temperature (T), pH, dissolved oxygen (DO), ammonium (NH_4_^+^), chlorophyll a (Chla), chemical oxygen demand (COD), turbidity, and oxidation–reduction potential (ORP) were measured at the site at 0.5 m intervals using a multi-parameter water quality analyzer (Hach Company). For the analysis of total nitrogen (TN) and total phosphorus (TP), water samples (approximately 40 mL) were collected into brown glass vials and acidified to pH < 2 with concentrated sulfuric acid. Water samples for physicochemical analysis of total suspended particles (TSP), TN, TP, NO_3_^−^, NO_2_^−^, dissolved organic carbon (DOC), and DIC were collected, transported, and analyzed according to previously established procedures (Zhang et al., 2018).

For each site, two filter pore sizes (0.22 μm and 3 μm) were used to collect microbial samples. Each sample (1.5 L) was filtered through a 3 μm polycarbonate membrane to separate particle-attached microorganisms (PA) from free-living microorganisms (FL, 0.22 μm) (Pan et al., 2022a,b). The surface sediment (0–5 cm) for microbial DNA extraction was collected from each sampling site and stored on dry ice in the field. At each site and during five different sampling stages, three microbial samples were collected simultaneously, representing particle-attached (PA), free-living (FL), and surface sediment (SS) fractions. All samples for microbial analysis were stored at −80 °C until further analysis.

### DNA extraction, high-throughput sequencing, and data processing

Total DNA from three replicates of 0.5 g sediment, PA, and FL microbial samples was extracted using a FastDNA SPIN Kit for Soil (MP Biomedicals, United States) according to the manufacturer’s instructions. Extracted DNA from SS, PA, and FL samples was amplified by PCR with barcoded primers targeting the 16S rRNA gene of bacteria and archaea: 338F (5′-ACTCCTACGGGAGGCAGCAG-3′)/806R (5′-GGACTACVSGGGTATCTAAT-3′). PCR reactions for each sample were performed in triplicate, and PCR amplification and purification of the product were conducted as described previously (Zhang et al., 2018).

The purified PCR amplicons (~400 bp) from each sample were sequenced using an Illumina HiSeq2500 platform with a PE250 model. Raw sequences of microbial 16S rRNA datasets were processed in QIIME2 (version 2021.2) (Bolyen et al., 2019). The DADA2 method was used to conduct sequence quality control, chimera removal, and feature table construction (Callahan et al., 2016).

Briefly, the *dada2 denoise-paired plugin* was applied with the following parameters: –*p-trim-left-f 19*, –*p-trim-left-r 20*, –*p-trunc-len-f 210,* and –*p-trunc-len-r 210.* The frequency of ASVs (amplicon sequence variants) was reported in the feature tables. The taxonomy of the representative prokaryotes was classified using the function *usearch-sintax* in USEARCH (v11.0.667) with an 80% confidence threshold against the SILVA v138 database (Edgar, 2016). Non-prokaryotic singletons and the ASVs with a frequency of *<*10 were removed from the feature tables. Alpha diversity indices (including observed richness, ASVs, Shannon, Simpson, Pielou, and Goods_coverage) were calculated using the R package “vegan.” All the 16S rRNA gene sequences obtained from this study have been deposited at the National Omics Data Encyclopedia (NODE, https://www.biosino.org/node/) under project OEP00006114.

Microbial metabolic potentials were predicted with PICRUSt2 based on 16S rRNA gene amplicon sequencing data (Douglas et al., 2020). The abundance table of the resulting KEGG Orthologs (KOs) was mapped to the KEGG pathway database (Ogata et al., 1999). The main metabolic pathways involved in the cycling of carbon (C), nitrogen (N), phosphorus (P), and sulfur (S) were selected for further analysis. The potential of each pathway was quantified by summing the relative abundances of all the KOs annotated with that specific pathway in each sample (Liu et al., 2020).

### Statistical analyses

Subsequent analyses were performed using R (V4.0.3) with appropriate packages. Differences in microbial diversity indices (SS, PA, and FL) in relation to environmental parameters across different stages of WSR were analyzed by one-way ANOVA, with a significance threshold set at a *p*-value of ≤0.05. To evaluate correlations between physiochemical factors and microbial α-diversity, as well as correlations among physiochemical factors, Pearson’s correlations were assessed, and Mantel tests were performed using the R package “vegan.”

A two-factor permutational multivariate analysis of variance (PERMANOVA) was employed to delineate the independent and interactive influences of temporal variation (WSR period) and spatial heterogeneity (sampling site) on microbial community structure. This analysis aimed to ascertain whether the WSR period had a significant effect on the microbial communities within the SS, PA, and FL fractions. Dissimilarity in community composition was quantified using Bray–Curtis distance matrices, and inferences were derived through 999 permutations, a methodology implemented within the R package “vegan” (Iqbal et al., 2023). Further assessment of microbial compositional disparities was conducted via principal coordinate analysis (PCoA), with PERMANOVA serving as the statistical framework to validate observed patterns, also executed using the “vegan” package. The distribution and overlap of microbial operational taxonomic units (OTUs) across the SS, PA, and FL sites at various stages of the WSR were visualized using Venn diagrams, generated with the “Venn Diagram” R package. Predictive functional profiling of the microbial communities in SS, PA, and FL across the WSR stages was achieved using PICRUSt, leveraging 16S rRNA gene amplicon sequencing data. Inter-stage functional differences were subsequently evaluated through one-way analysis of variance (ANOVA).

Molecular ecological networks (MENs) were constructed using the Random Matrix Theory (RMT) method (Yuan et al., 2021) to evaluate the differences in microbial interspecific interactions among SS, PA, and FL across four WSR stages in the study. Briefly, abundant OTUs (with a relative abundance of ≥0.05%) were used in the calculation to ensure reliable correlation networks, and only OTUs present in at least 50% of the samples were retained for network construction. All MENs were constructed based on Pearson correlations of log-transformed OTU abundances using the Molecular Ecology Network Analyses Pipeline (MENAP)^1^ (Deng et al., 2012). Pearson’s correlation coefficient |*R*| > 0.9 with *p* < 0.01 was used to construct the networks, where nodes represented microbial OTUs and edges indicated strong and significant correlations between nodes. Various network topological indices, such as the total number of nodes (*N*), total number of links (*L*), average degree, modularity, average path distance (PD), and average clustering coefficient (CC), were calculated to characterize the topological structure of the MENs (Wang S. et al., 2021; Liu et al., 2023). The constructed network visualization was conducted using Gephi 0.9.2 software (Bastian et al., 2009). To ascertain whether variations in network complexity were systematically influenced by differential diversity or sequencing depth, we conducted Pearson correlation analyses. These analyses examined the relationships between salient network metrics—specifically, the number of edges, modularity, and clustering coefficient—and both mean Shannon diversity and sequencing depth across all samples (Faust et al., 2015).

The relative importance of individual environmental factors on microbial community structure was evaluated by hierarchical partitioning analysis using the “rdacca.hp” package (Lai et al., 2022). Specifically, variance inflation factors (VIFs) were computed to check for collinearities among environmental variables. Variables with VIFs >10 were removed from subsequent hierarchical partitioning analysis until all VIFs were <10. The significance (*p* < 0.05) of the explanatory role of each environmental factor on microbial community structure was obtained through 999 permutation tests.

While hierarchical partitioning identified key environmental drivers, this correlation-based inference could not directly disentangle the underlying ecological processes governing community assembly. To explicitly quantify the relative contributions of deterministic and stochastic processes across different WSR stages, we conducted a null model-based framework. The analysis was performed independently for each of the five WSR periods to assess temporal dynamics. For each period, the β-nearest taxon index (βNTI) was computed for all pairwise sample comparisons. Deterministic processes were identified using a |βNTI| >2 threshold, where βNTI >+2 indicated variable selection and βNTI <−2 denoted homogeneous selection. For pairwise comparisons with |βNTI| <2, the subsequent metric employed was the Bray–Curtis–based Raup–Crick (RC) metric. Within this subset, |βNTI| <2 coupled with RC <−0.95 was interpreted as homogenizing dispersal, RC >+0.95 as dispersal limitation, and |RC| <0.95 as undominated processes. The relative dominance of each assembly process was quantified as the percentage of pairwise comparisons assigned to each category. All computations were performed using the “ape,” “iCAMP,” and “dplyr” R packages (Stegen et al., 2013).

LEfSe analysis (LDA score >2, *p* < 0.05) was performed to estimate significant variations, aiming to identify characteristic flora of SS, PA, and FL at different stages of WSR (Gao et al., 2024).

Moreover, the Pearson correlation was calculated between the dominant taxa (class level, relative abundance ≥0.05%) and the environmental factors measured, using the “vegan” and “ggplot” packages in R.

## Results

### Physicochemical variations among five different stages of WSR in the reservoir-river continuum of the Yellow River

The physicochemical characteristics exhibited distinct variations across the five designated stages of the water-sediment regulation (WSR) process within the reservoir-river continuum of the Yellow River, as detailed in Supplementary Figure S1. Water temperature fluctuated from 24.45 °C to 32.01 °C, with significantly elevated temperatures observed during the Post_WSR stage compared to both the Pre_WSR and Inter_WSR stages (*p* < 0.05) (Supplementary Figure S1A). pH levels ranged from 7.52 to 8.42, while turbidity varied from 17.05 to 77.80 NTU, reaching its maximum concentration in the Inter_WSR3 stage (77.80 NTU) (Supplementary Figures S1B,D).

Dissolved oxygen (DO), chlorophyll a (Chla), nitrate (NO_3_^−^), and total nitrogen (TN) concentrations exhibited a similar fluctuating pattern, ranging from 3.97 to 11.82 mg/L, 0.56 to 14.95 mg/L, 2.04 to 3.10 mg/L, and 0.06 to 1.88 mg/L, respectively. A significant decline in these parameters was noted during the Inter_WSR3 stage, followed by a statistically significant increase in the Post_WSR stage (*p* < 0.05) (Supplementary Figures S1C,E,H). Conversely, nitrite (NO_2_^−^) concentrations rose significantly to 0.17 mg/L during Inter_WSR3 and subsequently decreased to 0.02 mg/L in Post_WSR (*p* < 0.05). Likewise, dissolved inorganic carbon (DIC) showed a pronounced peak in Inter_WSR3 (40.61 mg/L), followed by a decline in Post_WSR (29.51 mg/L) (*p* < 0.05) (Supplementary Figures S1I,N).

TP concentrations exhibited a significant increase, rising from 0.06 mg/L during the Pre_WSR stage. This concentration peaked at 1.88 mg/L in the Inter_WSR2 stage, subsequently returning to levels comparable to those in the Pre_WSR stage (0.06–0.08 mg/L). Ammonium (NH_4_^+^) concentration increased initially to 0.67 mg/L and then decreased significantly to 0.12 mg/L (*p* < 0.05) compared to the Pre_WSR stage (Supplementary Figure S1F). The concentration of dissolved organic carbon (DOC) ranged from 8.76 mg/L to 11.39 mg/L, with no significant changes observed throughout the WSR process (*p* < 0.05) (Supplementary Figure S1M).

### Microbial alpha and beta diversity dynamics across WSR stages

A total of 12,498,869 high-quality sequence reads were generated from 120 samples collected across eight sampling sites, representing five distinct WSR stages. The average number of reads per sample was 104157.20 (±27703.71), with an average read length of 419 bp. Sequencing read counts varied slightly by sample location, with averages of 106,836,108,907, and 96,876 reads for microbial samples from the surface sediment (SS), particle-attached (PA), and free-living (FL) microorganisms, respectively.

Good’s coverage values were 1.0 for all samples, indicating that the sequence depth captured the majority of microbial taxa present. Alpha diversity metrics were calculated for all samples, and the results are summarized in Supplementary Table S1.

Overall, alpha diversity metrics demonstrated significant differences among sampling locations. The SS samples exhibited significantly higher average richness (2326.25 vs. 763.03; 2326.25 vs. 540.18), Shannon diversity (9.65 vs. 6.66; 9.65 vs. 6.39), and Pielou’s evenness (0.8632 vs. 0.6982; 0.8632 vs. 0.7071) indices compared to both the PA and FL locations (*p* < 0.05), with the exception of the Simpson index. However, no significant differences were observed between PA and FL for any alpha diversity index (*p* > 0.05), despite a trend showing higher richness and Shannon diversity values in PA compared to FL (763.03 vs. 540.18) (Supplementary Table S1).

**Figure 2:**
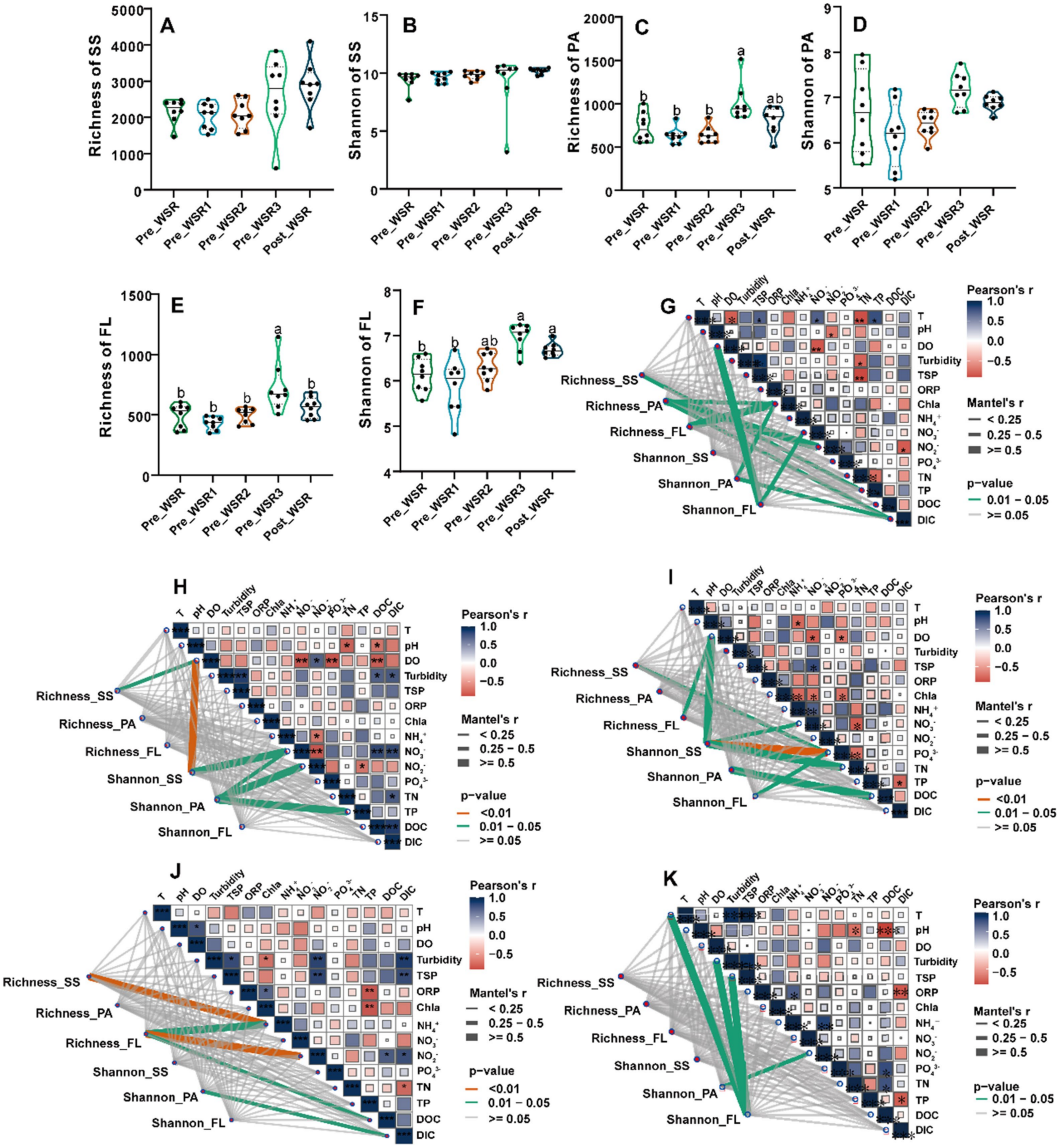
Microbial alpha-diversity indices and their correlations with environmental factors across different WSR stages in the study. **(A–F)** Comparison of richness and Shannon indices for surface sediment (SS), particle-attached (PA), and free-living (FL) microorganisms. Different superscript letters indicate significant differences (*p* < 0.05), while identical or unmarked letters indicate no significant differences (*p* > 0.05) (one-way ANOVA). **(G–K)** Pearson correlations between richness and Shannon indices of SS, PA, and FL and environmental factors, determined using the Mantel test analysis. **G–K** display the results of Mantel tests, illustrating the correlations between the Shannon and Richness indices of the SS, PA, and FL microbial communities and environmental factors across five periods: Pre_WSR, Inter_WSR1, Inter_WSR2, Inter_WSR3, and Post_WSR. The thickness of the connecting lines represents the magnitude of the Mantel correlation coefficient, while the color indicates the level of statistical significance: orange denotes *P* < 0.01, green indicates 0.01 < *P* < 0.05, and gray represents non-significant correlationsn (*P* ≥ 0.05). Additionally, the correlations among the environmental factors within each period are presented in the same subfigures. Positive and negative Pearson correlations are distinguished by red and blue colors, respectively, with significance levels marked as follows: “***”, *P* < 0.001; “**”, *P* < 0.01; and “*”, *P* < 0.05.

Detailed analysis revealed similar temporal trends in richness and Shannon diversity indices across SS, PA, and FL samples during the WSR process (Figures 2A–F). Compared to the Pre_WSR samples, both richness and Shannon diversity increased in the Post_WSR stage for SS (2175.75 vs. 2884.75; 9.41 vs. 10.17), PA (729.88 vs. 801.38; 6.69 vs. 6.88), and FL (491.13 vs. 560.50; 6.13 vs. 6.69). Interestingly, both PA (1009.63; 7.17) (Figures 2C,D) and FL (725.13; 6.97) (Figures 2E,F) showed statistically significant increases (*p* < 0.05) in richness and Shannon diversity to maximal values during the Inter_WSR3 stage, while SS did not. There was no significant difference in those indices compared to the Post_WSR samples (*p* > 0.05).

Two-factor PERMANOVA showed that, after controlling for variation across sampling sites, the WSR period significantly shaped microbial communities in all three compartments (PA: *p* = 0.001; FL: *p* = 0.001; SS: *p* = 0.001). Notably, the WSR stage exerted the most substantial influence on sediment microbial communities, explaining the highest proportion of variance (SS, *R*^2^ = 38.5%). The sampling site effect was also significant (*p* = 0.003; Table 1). Crucially, the WSR stage effect remained significant even after controlling for spatial variation, underscoring its independent impact.

**Table 1:** Two-factor PERMANOVA analysis revealed the effects of temporal (five WSR stages) and spatial (eight sampling sites within each WSR stage) variations on microbial community structure.

Microbial-type	Factor	*R*^2^	*p*-value
PA	Stages of WSR	0.158634	0.001
PA	Sampling Site	0.244915	0.001
FL	Stages of WSR	0.245445	0.001
FL	Sampling Site	0.315308	0.001
SS	Stages of WSR	0.384566	0.001
SS	Sampling Site	0.179415	0.003

**Figure 3:**
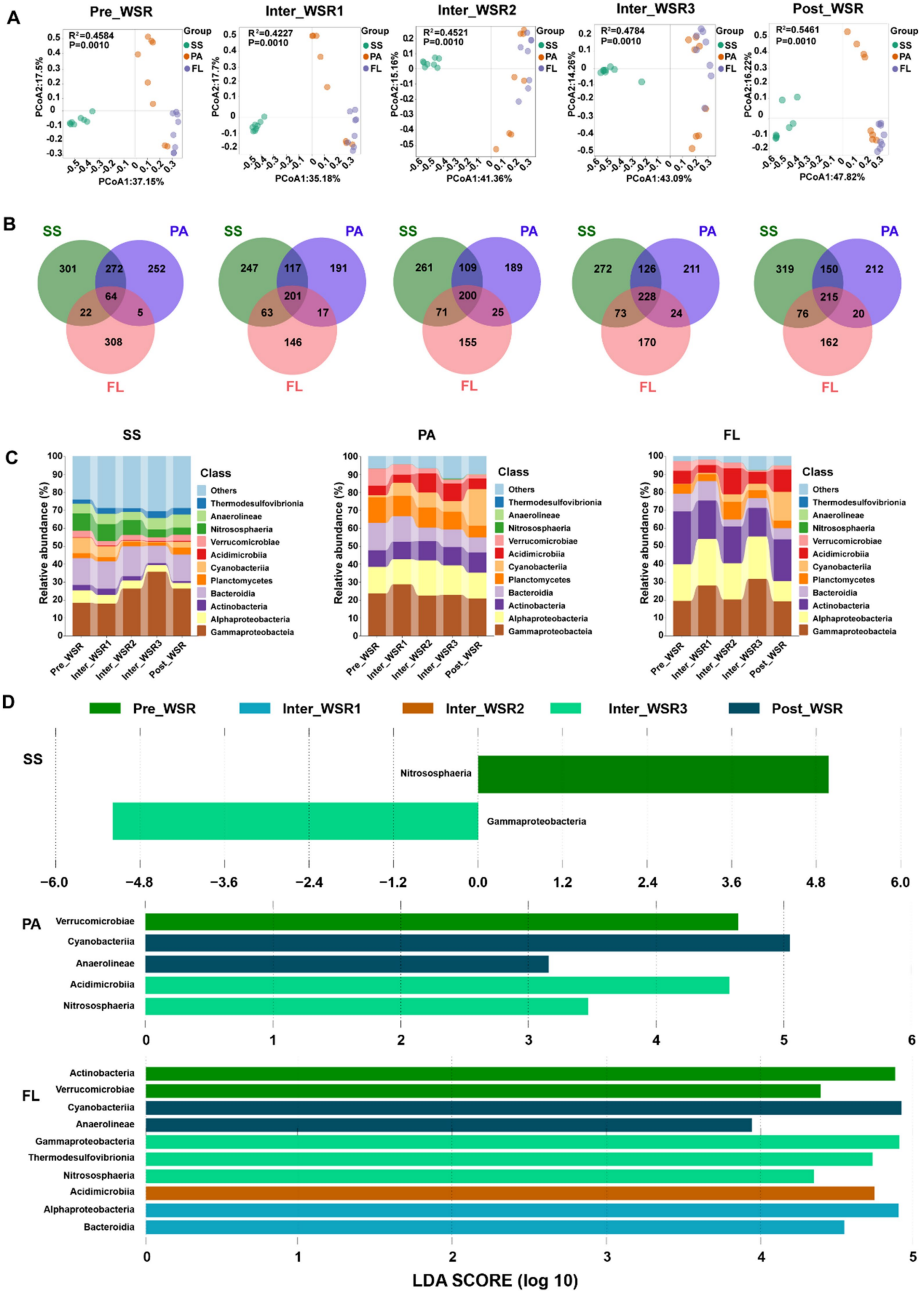
SS, PA, and FL-related microbial community variations at different stages of WSR. **(A)** PCoA with PERMANOVA (based on Bray–Curtis distance) analysis indicated significant differences (*p* < 0.05) in SS-, PA-, and FL-related microbial communities during the process of WSR. **(B)** Venn analysis shows the unique and the shared OTUs among SS, PA, and FL microorganisms during the process of WSR. **(C)** Microbial community composition changes (in class level, relative abundance ≥1%) during the process of WSR. **(D)** Microbial groups (class level) with significant differences selected by the LEfSe analysis (LDA score ≥2) during the process of WSR.

Beta diversity analyses, performed using principal coordinates analysis (PCoA) in combination with PERMANOVA, confirmed significant differences (*p* < 0.001) in microbial community structure among SS, PA, and FL throughout the WSR process (Figure 3A). Specifically, the microbial community composition of SS samples exhibited significant divergence from PA and FL, while the communities of PA and FL showed greater structural similarity. This differentiation was particularly pronounced during the Inter_WSR2 and Inter_WSR3 stages (Figure 3A).

Furthermore, Venn diagram analysis of operational taxonomic units (OTUs) showed that the number of shared OTUs among SS, PA, and FL gradually increased from the Inter_WSR1 stage (201) to the Inter_WSR3 stage (208), compared to the Pre_WSR stage (64), followed by a slight decrease in the Post_WSR stage (Figure 3B). The number of unique OTUs for SS, PA, and FL showed different patterns over the WSR process. Compared to the Pre_WSR stage, the number of SS-specific OTUs (301 vs. 309) increased overall. The number of PA-specific OTUs (252 vs. 212) decreased slightly, whereas FL-specific OTUs (308 vs. 162) decreased most notably during the Post_WSR stage (Figure 3B).

### Microbial community structure variation during WSR

Across all samples collected throughout the WSR process, the dominant microbial classes (with an average relative abundance of ≥0.5%) exhibited a high degree of similarity among the sedimentation site (SS), plunge area (PA), and flowing reach (FL). These core classes included *Gammaproteobacteria* (17.09–35.69%), followed by *Alphaproteobacteria* (3.17–25.85%), *Actinobacteria* (4.01–16.72%), *Bacteroidia* (0.98–29.42%), *Planctomycetes* (1.88–14.18%), *Cyanobacteriia* (0.13–20.27%), *Acidimicrobiia* (1.88–14.18%), *Verrucomicrobiae* (0.77–9.49%), *Nitrososphaeria* (0–9.62%), *Anaerolineae* (0–7.26%), and *Thermodesulfovibrionia* (0–3.74%) (Figures 3B,C and Supplementary Tables S2–S4).

Despite the overall similarity in community composition, the relative abundance of these dominant classes varied among SS, PA, and FL. For example, *Gammaproteobacteria* (24.92%) and *Bacteroidia* (14.29%) were most abundant in SS, followed by FL (23.74 and 7.72%) and PA (23.66 and 11.02%). Conversely, *Planctomycetes* (10.62%) and *Cyanobacteria* (8.57%) exhibited the highest relative abundance in PA, followed by FL (5.51 and 4.91%) and SS (2.66 and 3.76%). *Alphaproteobacteria* (20.28%) and *Actinobacteria* (22.06%) were most abundant in FL, followed by PA (15.85 and 10.24%) and SS (4.46 and 2.18%) (Figures 3B,C and Supplementary Tables S2–S4).

Furthermore, the dominant groups and their abundance changed, showing distinct trends across the different WSR stages. For instance, from Pre_WSR to Post_WSR, the abundance of *Gammaproteobacteria* decreased in all three locations, but the temporal pattern varied among them. Specifically, SS-*Gammaproteobacteria* (35.69%) increased from the Pre_WSR to the Inter_WSR3, with its abundance declining to 26.31% in Post_WSR. PA-*Gammaproteobacteria* peaked in the Inter_WSR2 (28.67%), then decreased to 20.82% in Post_WSR. The abundance of FL-*Gammaproteobacteria* fluctuated throughout WSR, reaching peaks in the Inter_WSR1 (28.07%) and Inter_WSR3 (31.65%) stages and returning to levels similar to Pre_WSR in Post_WSR.

To elucidate the distribution of abundant taxa at different stages of WSR across SS, PA, and FL, LEfSe analysis (LDA score >2, *p* < 0.05) was conducted to identify significant variations. The results showed that the number of significantly different microbial classes was highest in FL (10 classes), followed by PA (five classes), and SS (two classes). The enriched classes varied among WSR stages (Figure 3D). In SS, *itrososphaeria* and *Gammaproteobacteria* were enriched in Pre_WSR and Inter_WSR3, respectively. In PA, *Verrucomicrobiae* were enriched in Pre_WSR; *Acidimicrobiia* and *Nitrososphaeria* were enriched in Inter_WSR3; and *Cyanobacteriia* and *Anaerolineae* were enriched in Post_WSR. In FL, *Actinobacteria* and *Verrucomicrobiae* were enriched in Pre_WSR; *Alphaproteobacteria* and *Bacteroidia* were enriched in Inter_WSR1; *Acidimicrobiia* were enriched in Inter_WSR2; *Gammaproteobacteria*, *Thermodesulfovibrionia*, and *Nitrososphaeria* were enriched in Inter_WSR3; and *Cyanobacteriia* and *Anaerolineae* were enriched in Post_WSR (Figure 3D).

In detail, *Nitrososphaeria* was significantly enriched in SS during Pre_WSR. Its abundance also increased in both PA and FL in Inter_WSR2 (Figure 3C). *Gammaproteobacteria* in SS was significantly enriched in Inter_WSR3 compared to other WSR stages. Moreover, *Cyanobacteriia* and *Anaerolineae* were enriched in both PA and FL in Post_WSR (Figure 3C and Supplementary Table S3).

### Microbial ecological function changes during WSR

**Figure 4:**
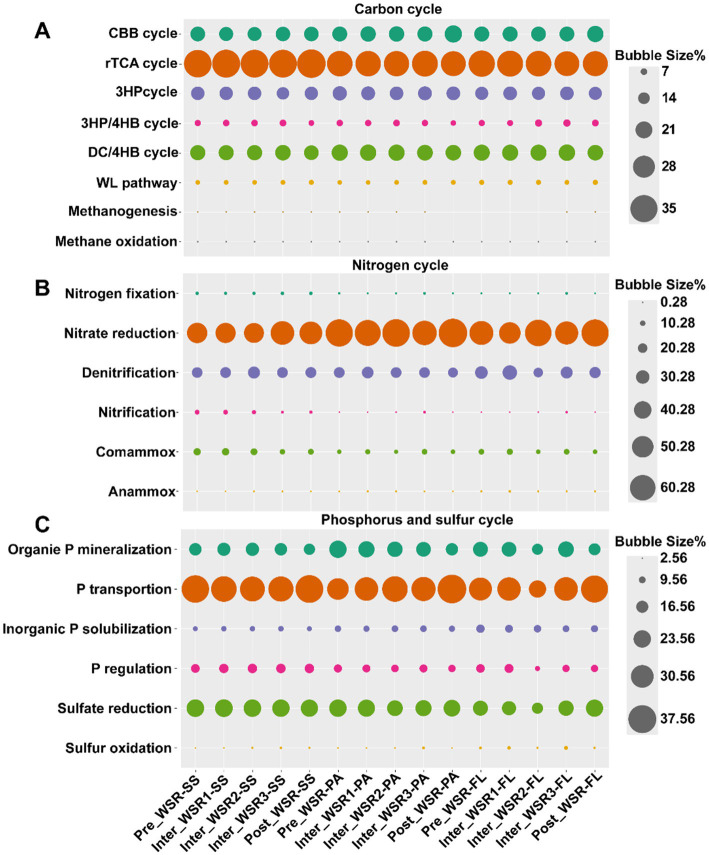
Relative abundance of microbial function shifts across WSR stages (predicted by PICRUSt based on 16S rRNA high-throughput sequencing data). **(A)** Carbon cycles: CBB cycle, Calvin–Benson–Bassham cycle; rTCA cycle, reductive tricarboxylic acid cycle; 3HP cycle, 3-hydroxypropionic acid cycle; 3HP/4HB cycle, 3-hydroxypropionic acid/4-hydroxybutanoic acid cycle; DC/4HB cycle, dicarboxylic acid/4-hydroxybutyrate cycle; WL pathway, Wood–Ljungdahl pathway. **(B)** Nitrogen cycles. **(C)** Phosphorus and sulfur cycles.

PICRUSt-based functional prediction indicated that microorganisms in SS, PA, and FL communities contributed to carbon, nitrogen, phosphorus, and sulfur cycling in the turbid Yellow River ecosystem. Underwater sediment regulation (WSR) caused changes over time in the abundance of genes related to these ecological functions (Figure 4; Supplementary Figures S2–S4).

In the carbon cycle, the reductive tricarboxylic acid (rTCA) cycle was the most abundant (26.99–29.98%), highest in SS (29.98%), and slightly lower in PA (27.75%) and FL (27.69%) (Figure 4). rTCA remained stable in SS and PA but declined in FL (*p* < 0.05, Supplementary Figure S2B). The dicarboxylate/4-hydroxybutyrate (DC/4HB) cycle was lowest in SS (20.64%) and decreased significantly in PA and FL (*p* < 0.05, Supplementary Figure S2F). The Calvin–Benson–Bassham (CBB) cycle (18.84–23.03%) was lower in SS (19.45%) than in PA (20.72%) and FL (20.22%), remaining stable in SS but increasing significantly in PA and FL (*p* < 0.05, Supplementary Figure S2A).

For nitrogen cycling, nitrate reduction dominated (46.32–67.71%), with the highest rate in PA (63.30%) compared to SS (49.93%) and FL (57.56%). Denitrification followed (21.44–32.66%), being more abundant in FL (32.66%) than in SS (24.23%) or PA (23.46%) (Figure 4). Across WSR, neither process differed significantly, but at Inter_WSR3, nitrate reduction decreased in PA and FL, while denitrification increased. Comammox (7.08–15.05%) was also abundant, highest in SS (13.05%), followed by FL (10.13%) and PA (8.66%). During WSR, comammox declined in SS (*p* < 0.05), but increased in PA and FL at Inter_WSR3. Other detected pathways included anammox, nitrification, and nitrogen fixation (Supplementary Figure S3).

For phosphorus cycling, phosphorus transport dominated (29.19–38.41%), highest in SS (35.22%), then in PA (33.28%), and in FL (32.70%) (Figure 4). During WSR, transport in PA declined at Inter_WSR3 (*p* < 0.05) before recovering, while in FL it increased steadily. Organic phosphorus mineralization (15.55–23.50%) was relatively abundant, higher in SS (12.92%) than in PA (9.18%) and in FL (10.59%) but declined significantly in PA and FL, with a temporary increase at Inter_WSR3 in FL (*p* < 0.05, Supplementary Figure S4A). Phosphorus regulation (10.27–13.17%) peaked at Inter_WSR2 before declining, while solubilization (7.23–11.72%) decreased steadily (Supplementary Figure S4).

In the sulfur cycle, sulfate reduction was predominant (19.36–24.07%), highest in SS (23.75%), followed by PA (22.62%) and FL (21.13%). Sulfate oxidation was next, more abundant in FL (4.55%) than in SS (3.25%) or PA (3.13%), and it increased continuously in PA and FL during WSR (*p* < 0.05) (Figure 4).

### Features of microbial molecular networks in SS, PA, and FL during WSR

**Figure 5:**
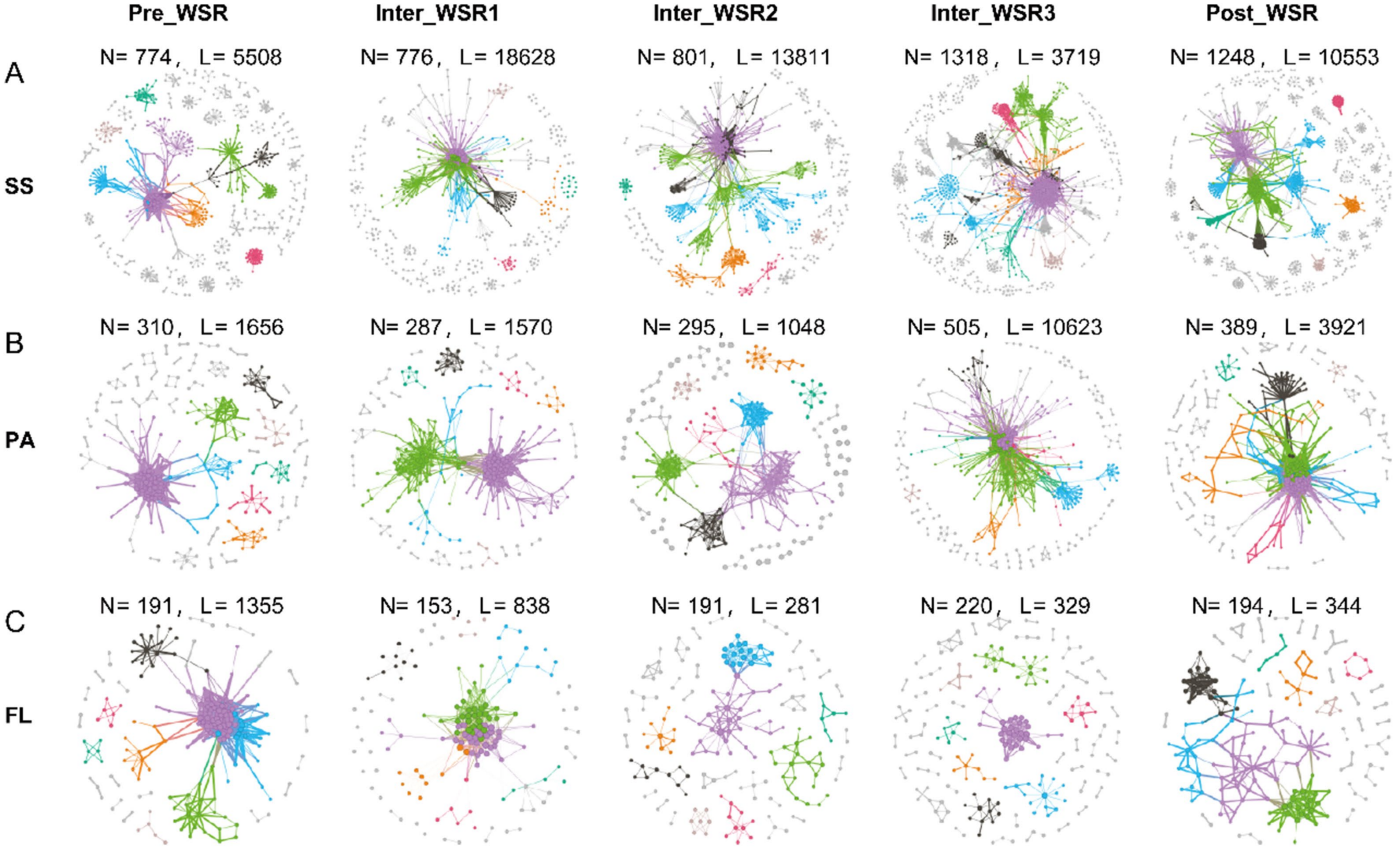
Molecular ecological networks (MENs) of SS, PA, and FL-related microbial communities at different stages of WSR (N, nodes; L, links) based on the OTU table from 16S rRNA sequencing data. **(A–C)** The microbial networks represent random matrix theory (RMT)-based correlation models derived from SS, PA, and FL samples at each WSR stage in the study. Nodes represent ASVs, and links between nodes represent significant correlations (either positive or negative). The node size is proportional to its degree, and colors indicate different modules.

Microbial molecular networks (MENs) were constructed based on OTUs from the Pre_WSR, Inter_WSR1, Inter_WSR3, and Post_WSR stages for SS, PA, and FL samples to evaluate potential interactions within the microbial communities (Figure 4 and Table 1). Overall, the MENs in SS exhibited the highest network complexity, as indicated by the greatest values across several metrics (nodes: 774–1,318; links: 3,719–18,628; positive links/negative links (NL/PL): 0.86–2.24; average degree: 0.22–48.01; modularity: 0.11–0.73; average path distance (PD): 3.25–5.6; average clustering coefficient avgCC: 0.12–0.25). PA displayed intermediate network complexity (nodes: 287–505; links: 1,048–10,623; NL/PL: 1.07–2.73; average degree: 7.11–42.07; modularity: 0.11–0.77; PD: 3.11–4.41; avgCC: 0.18–0.23), while FL had the lowest complexity (nodes: 153–220; links: 281–1,355; N/P: 0.81–2.07; average degree: 2.94–10.95; modularity: 0.22–0.83; PD: 2.12–5.01; avgCC: 0.12–0.21) (Figure 5 and Table 2).

**Table 2:** Topological properties of SS, PA, and FL at different stages of WSR (mean values from eight sampling sites, with three replicates for each of PA, FL, and SS at each stage; *n* = 24).

Topological properties	Pre_WSR			Inter_WSR1			Inter_WSR2			Inter_WSR2			Post_WSR		
	SS	PA	FL	SS	PA	FL	SS	PA	FL	SS	PA	FL	SS	PA	FL
Numbers of nodes (*N*)	774	310	191	776	287	153	801	295	191	1,318	505	220	1,248	389	194
Numbers of links (*L*)	5,508	1,656	1,355	18,628	1,570	838	13,811	1,048	281	3,719	10,623	329	10,553	3,921	344
Positive links (PL)	1,700	444	468	8,826	553	295	6,245	361	128	2,002	4,261	107	3,103	1,893	190
Negative links (NL)	3,808	1,212	887	9,802	1,017	543	7,566	687	153	1,717	6,362	222	7,450	2,028	154
NL/PL	2.24	2.73	1.90	1.11	1.84	1.84	1.21	1.90	1.20	0.86	1.49	2.07	2.40	1.07	0.81
Average degree	14.23	10.68	14.19	48.01	10.94	10.95	34.48	7.11	2.94	0.22	42.07	2.99	16.91	20.16	3.55
Modularity	0.32	0.30	0.20	0.11	0.67	0.22	0.16	0.77	0.83	0.73	0.11	0.77	0.49	0.17	0.77
Average path distance (PD)	5.01	3.97	3.35	3.25	3.81	3.20	4.28	4.41	3.28	5.60	3.11	2.12	4.80	3.29	5.01
Average clustering coefficient (avgCC)	0.13	0.18	0.21	0.25	0.22	0.19	0.23	0.19	0.15	0.22	0.24	0.12	0.12	0.23	0.14

*N*, number of nodes; L, number of links; PL, positive links; NL, negative links; NL/PL, positive links/negative links; PD, average path distance; avgCC, average clustering coefficient.

Specifically, the modularity (0.32–0.73) and the number of nodes (774–1,318) in the SS networks increased from Pre_WSR to Inter_WSR3, while the two metrics decreased to 0.49 and 1,248, respectively, in the Post_WSR. Similar trends were observed in PA, where the modularity (0.30–0.77) and the number of nodes (310–505) increased from Pre_WSR to Inter_WSR3 before decreasing to 0.17 and 389, respectively, in the Post_WSR. In contrast, FL exhibited relatively low values for modularity (0.20–0.22) and the number of nodes (191) in Inter_WSR1 and Inter_WSR2. These values increased in the subsequent stages, reaching a maximum of 0.77–0.83 and 194–220, respectively, from Inter_WSR3 (Figure 5 and Table 2).

Compared to Pre_WSR, both the number of links and the average clustering coefficient (avgCC) of SS networks increased during Inter_WSR1 (links: 18,628; avgCC: 0.22), decreased to a minimum during Inter_WSR3 (3,719), and subsequently rose to 10,553 links in Post-WSR. The average clustering coefficient (avgCC) exhibited relatively stable values from Inter_WSR1 to Post_WSR (Figure 5 and Table 2). The number of links in PA also exhibited a fluctuating upward trend from Pre_WSR (1,656) to Inter_WSR3 (10,623), followed by a decrease to 3,912 in the Post_WSR. Conversely, the number of links in FL showed a downward fluctuating trend, decreasing from Pre_WSR (1,355) to Inter_WSR3 (329) and then rising to 344 in the Post_WSR.

Moreover, compared to the Pre_WSR stage, the N/P (0.86) and average degree (0.22) values of SS networks consistently decreased, reaching their lowest values during Inter_WSR3. Both Post_WSR values (N/P: 2.40; average degree: 16.91) of SS returned to levels similar to those of Pre_WSR (N/P: 2.24; average degree: 14.23). In contrast, the path diameter (PD) of SS networks reached the highest value in Inter_WSR3, then decreased to a value of 4.80 in Post_WSR, which was similar to that of Pre_WSR (5.01). The N/P value of PA showed a fluctuating decrease from Pre_WSR (2.73) to Post_WSR (1.07), reaching its lowest level in Post_WSR, while the average degree of PA reached its highest value of 42.07 in Inter_WSR3. The N/P value of FL reached its maximum of 2.07 in Inter_WSR3 and then decreased to 0.81 in Post_WSR. In contrast, the path diameter (PD) of FL reached its lowest value (2.12) in Inter_WSR3 and then increased to 5.01 in Post_WSR (Figure 5 and Table 2).

The correlation analyses revealed that, while the number of edges showed a significant positive correlation with Shannon diversity (*R* = 0.766, *p* = 0.001), the fundamental topological properties of modularity (*R* = −0.232, *p* = 0.405) and clustering coefficient (*R* = −0.022, *p* = 0.939) demonstrated no significant relationship with diversity. Importantly, none of the three-network metrics correlated significantly with sequencing depth (all *p* > 0.5) (Supplementary Table S7).

### Influence of physicochemical factors on the microbial distribution during WSR

**Figure 6:**
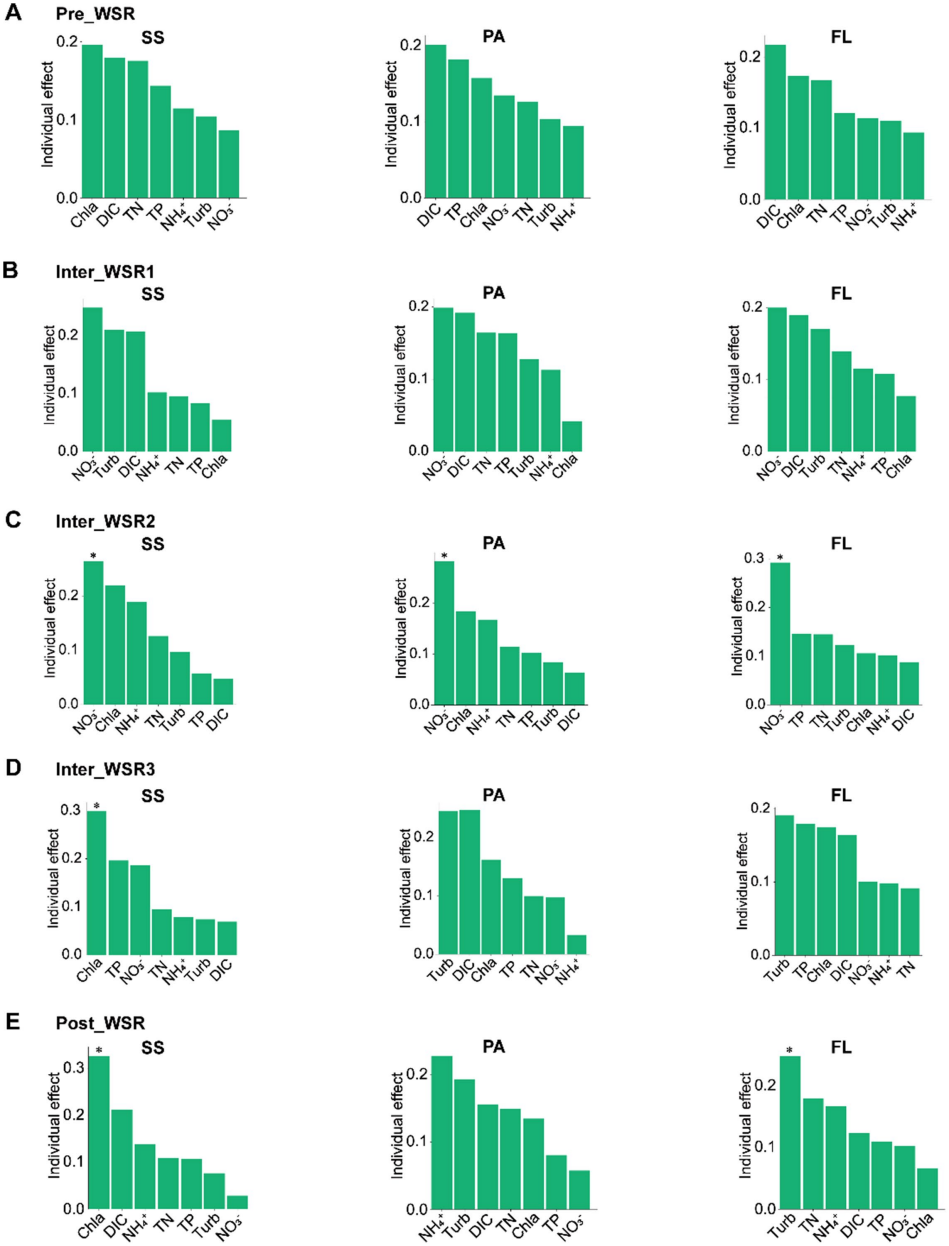
HP (hierarchical partitioning) analysis showing the contribution of physicochemical factors to SS, PA, and FL-related microbial communities at different stages of WSR. **(A–E)** The variance inflation factor (VIF) was calculated to retain the physicochemical factors with low collinearity. ^*^p < 0.05, which shows that this physicochemical factor contributes significantly to the microbial community structure (permutation test). “Turb” is the abbreviation of turbidity.

The relationships between microbial communities and physicochemical factors were investigated across the WSR process. Changes in microbial richness and Shannon diversity in SS, PA, and FL exhibited varying correlations with physicochemical factors (Figures 2G–K). Additionally, the contribution of key physicochemical parameters (Chla, DIC, TN, TP, NH_4_^+^, turbidity, and NO_3_^−^) to the microbial community structure in SS, PA, and FL shifted throughout the WSR stages. Furthermore, even within the same WSR stage, these important physicochemical factors differed in their influence on the microbial community structure of SS, PA, and FL (Figure 6).

Mantel test results indicated that physicochemical factors showed stronger correlations with microbial richness and Shannon diversity in Pre_WSR, Inter_WSR2, and Inter_WSR3 compared to Inter_WSR1 and Post_WSR (Figures 2G–K). Specifically, the richness of SS was correlated with TN, pH, NO_3_^−^, and Chla (*p* < 0.01) in Pre_WSR, Inter_WSR1, Inter_WSR2, and Inter_WSR3, respectively (Figures 2G–K). Shannon diversity in SS correlated with pH (*p* < 0.01) and NH_4_^+^ in Inter_WSR1 and correlated with pH, NH_4_^+^, and NO_2_^−^ (*p* < 0.01) in Inter_WSR2. Regarding PA, richness was only correlated with oxidation–reduction potential (ORP), NO_3_^−^, and dissolved organic carbon (DOC) in Pre_WSR, while Shannon diversity was correlated with ORP and DOC in Pre_WSR and with NH_4_^+^, NO_3_^−^, and TN in Inter_WSR1. From Inter_WSR2 to Post_WSR, Shannon diversity in PA was solely correlated with TP, DOC, and NO_3_^−^, respectively (Figures 2G–K). FL richness was correlated with ORP and pH in Pre_WSR and Inter_WSR3, respectively. In Inter_WSR3, richness also correlated with Chla and TP (*p* < 0.01). Shannon diversity in FL was correlated with pH and NH_4_^+^ in Pre_WSR, only NO_2_^−^ in Inter_WSR2, but correlated with T, DO, and turbidity in Post_WSR (Figures 2G–K).

Hierarchical partitioning analysis revealed that, in the Pre_WSR, Chla was the largest contributor to SS (0.20), followed by DIC (0.18) and TN (0.18). However, DIC was the primary contributor to PA (0.20) and FL (0.22) during this stage. In Inter_WSR1, NO_3_^−^ became the greatest contributor to SS, PA, and FL, with turbidity and DIC acting as the second major contributors (Figure 6A). Inter_WSR2 saw nitrate become the most significant physicochemical factor, contributing significantly (*p* < 0.05) to SS (0.26), PA (0.28), and FL (0.29). Furthermore, Chla and NH_4_^+^ also exhibited high contributions to SS and PA. For FL, the second and third highest contributors were TP and TN during the Inter_WSR2 stage (Figure 6B). In Inter_WSR3, a period of sand regulation, Chla became the most significant contributor to SS (0.30, *p* < 0.05), while turbidity became the dominant contributor to both PA and FL. Conversely, TP and NO_3_^−^ were key contributors to SS, DIC, and Chla were important contributors to PA, and TP and NO_3_^−^ were also important contributors to FL (Figure 6C). At the end of WSR (Post_WSR), Chla remained the greatest contributor to SS (0.33, *p* < 0.05). However, NH_4_^+^ became the dominant contributor to PA (0.23), and turbidity dominated FL (0.25, *p* < 0.05) (Figures 6D).

To move beyond merely identifying key environmental drivers and to explicitly elucidate the ecological mechanisms through which these factors shape community structure, we performed a null model analysis of community assembly processes. The null model analyses revealed distinct ecological assembly processes governing SS, PA, and FL microbial communities across the five WSR stages (Supplementary Figure S5). In SS communities, most pairwise comparisons yielded |βNTI| <2 across all stages. However, a notable number of comparisons in Pre_WSR exhibited βNTI <−2 (Supplementary Figure S5A). Consistent with this, the quantitative estimation of ecological processes identified homogeneous selection as the dominant assembly mechanism in Pre_WSR. In subsequent stages, community assembly shifted to being primarily governed by undominated processes (Supplementary Figure S5B). For PA communities, βNTI values also largely fell within the |βNTI| <2 range, though several comparisons in Pre_WSR showed βNTI >+2 (Supplementary Figure S5C). Ecological process partitioning indicated substantial contributions of homogenizing dispersal during Inter_WSR1 and Inter_WSR3. Throughout the WSR stages, the combined influence of undominated processes and homogenizing dispersal represented the principal assembly mechanisms in PA communities (Supplementary Figure S5D). In FL communities, nearly all βNTI values across the five stages were within the |βNTI| <2 threshold (Supplementary Figure S5E). Accordingly, undominated processes overwhelmingly dominated the assembly, accounting for the highest relative proportion among all three habitats in each stage. Deterministic processes, namely homogeneous and variable selection, had minimal influence (Supplementary Figure S5F).

### Correlations between microbial community and physiochemical variables during WSR

**Figure 7:**
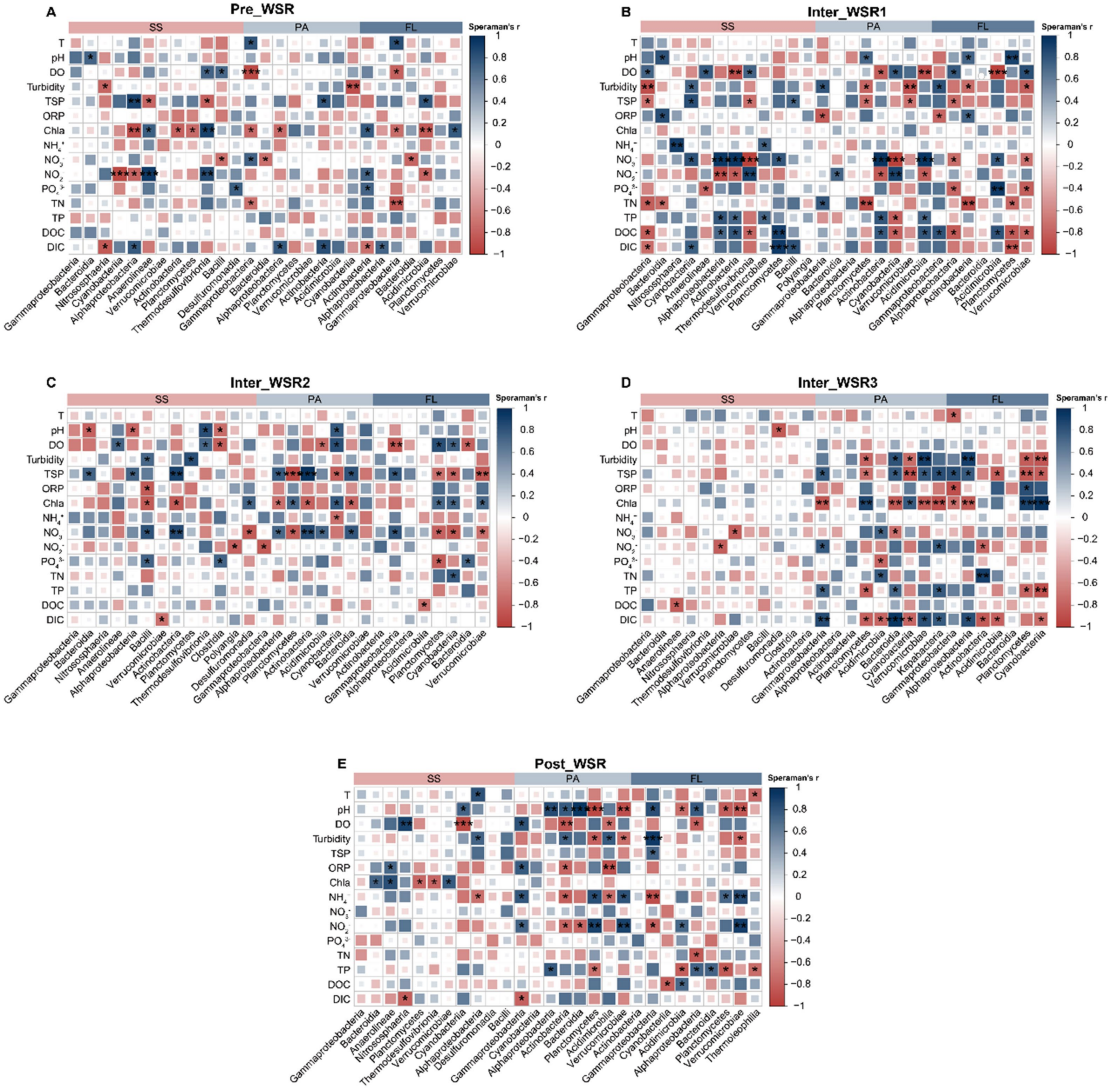
Spearman correlation between the relative abundance of dominant taxa (at the class level, relative abundance >1%) and the measured physicochemical variables for the SS, PA, and FL-related microorganisms in different stages of WSR. **(A)** Pre_WSR. **(B)** Inter_WSR1. **(C)** Inter_WSR2. **(D)** Inter_WSR3. **(E)** Post_WSR. ^***^p < 0.001, ^**^p < 0.01, and ^*^p < 0.05.

Pearson correlation analysis was employed to assess the relationships between the relative abundance of dominant microbial taxa (at the class level, with a relative abundance of >1%) and various physicochemical factors throughout the WSR process. The results revealed that many taxa were significantly correlated with dissolved oxygen (DO), turbidity, total suspended particles (TSP), chlorophyll a (Chla), NO_3_^−^, NO_2_^−^, total nitrogen (TN), and total phosphorus (TP) (Figure 7).

In the Pre_WSR stage, the dominant taxa in SS were most significantly (*p* < 0.05) influenced by TSP, Chla, NO_3_^−^, and NO_2_^−^, while PA and FL showed fewer correlations with these factors compared to SS (Figure 7A). During Inter_WSR1 and Inter_WSR2, a greater number of physicochemical factors exhibited significant (*p* < 0.05) correlations with the microbial communities in SS, PA, and FL. In contrast, during Inter_WSR3, the PA and FL communities exhibited stronger correlations with turbidity, TSP, Chla, TP, and DIC compared to the SS community (Figures 7A–C). As WSR concluded (Post_WSR), correlations were observed between certain dominant taxa and DO, oxidation–reduction potential (ORP), and Chla. Simultaneously, pH, DO, ORP, NH_4_^+^, NO_2_^−^, TP, and dissolved organic carbon (DOC) emerged as key physicochemical factors exhibiting significant correlations with the dominant taxa in PA and FL (Figure 7D).

Furthermore, the degree of influence of physicochemical factors on microbial communities varied among SS, PA, and FL throughout the WSR stages (Figure 7). During Pre_WSR, *Alphaproteobacteria*, *Anaerolineae*, and *Thermodesulfovibrionia* in SS were most sensitive to changes in the physicochemical environment. Specifically, *Alphaproteobacteria* displayed a positive correlation with TSP and DIC (*p* < 0.05) and a negative correlation with Chla and NO_2_^−^ (*p* < 0.01). *Anaerolineae* and *Thermodesulfovibrionia* displayed positive correlations with Chla and NO_2_^−^ (*p* < 0.05) and negative correlations with TSP (*p* < 0.05) (Figure 7A). *Gammaproteobacteria* were the most vulnerable taxa in PA and FL, showing positive correlations with temperature (T) (*p* < 0.05) and negative correlations with DO, Chla, and TN (*p* < 0.05). Furthermore, *Actinobacteria* in FL showed positive correlations with Chla, NO_2_^−^, and PO_4_^3−^ and a negative correlation with DIC. Compared to Pre_WSR, the number of dominant classes in Inter_WSR1 that were sensitive to the physicochemical factors increased in SS (e.g., *Alphaproteobacteria*, *Gammaproteobacteria*, *Cyanobacteria*, *Actinobacteria*), PA (e.g., *Planctomycetes*, *Cyanobacteria*, *Actinobacteria*), and FL (e.g., *Alphaproteobacteria*, *Gammaproteobacteria*, *Acidimicrobiota*) (Figure 7B). In contrast, the number of significant correlations between the dominant classes of SS (*Cyanobacteria*) and the physicochemical factors became limited in Inter_WSR3 (Figure 7D). However, as the WSR process concluded (Post_WSR), certain classes in SS regained correlations with physicochemical factors, such as the positive correlations between *Anaerolineae* and Chla and ORP (*p* < 0.05). Simultaneously, the responses of some taxa in PA and FL to the physicochemical factors became weaker. For example, *Bacteroidia* in PA during Inter_WSR3 exhibited positive correlations with DIC, turbidity, TSP, and TP, while showing negative correlations with NO_3_^−^. In contrast, during the Pre_WSR, *Bacteroidia* in PA demonstrated a significant positive correlation with pH (*p* < 0.05) and a significant correlation with NO_2_^−^ (*p* < 0.05). A similar pattern was observed for *Cyanobacteria* in FL during Inter_WSR3, which correlated positively with Chla but negatively with turbidity, TP, TSP, and DIC. However, in the Post_WSR, *Cyanobacteria* only showed a negative correlation with DOC (Figures 7D,E).

## Discussion

### Microbial community responses to WSR: stage-specific shifts and legacy effects

Microbial communities in the Yellow River exhibited stage-specific successions in response to water-sediment regulation (WSR), with the most pronounced disruption occurring during the sediment-regulation stage (Inter_WSR3). This phase was characterized by extreme hydraulic turbulence, sediment resuspension, and pulsed nutrient fluxes (e.g., NO₃^−^, DIC), which collectively restructured microbial interactions and community composition (Figure 3A). Notably, microbial α-diversity (richness and evenness) initially increased during WSR but reverted to pre-disturbance levels in Post-WSR (Figures 2A–F), suggesting a transient resilience of taxonomic diversity. However, this recovery masked underlying shifts in β-diversity, as revealed by principal coordinate analysis (PCoA), which showed persistent divergence among surface sediment (SS), particle-attached (PA), and free-living (FL) microbial communities throughout WSR (Figure 3A). Such divergence reflects niche differentiation driven by stage-specific physicochemical gradients, such as light availability, nutrient concentrations, and sediment-water interactions (Xia et al., 2013; Pan et al., 2022a, 2022b).

Molecular ecological network analysis (MENs) further illuminated the destabilizing effects of WSR on microbial interactions. Network complexity (nodes, links, modularity) peaked during Inter_WSR3 but failed to fully recover post-disturbance (Table 1 and Figure 5). For instance, the modularity of SS microbial networks dropped from 0.73 during Inter_WSR3 to 0.49 in Post-WSR, indicating a collapse of the modular community structure critical for functional redundancy (Barberán et al., 2012). This legacy effect implies that even short-term WSR disturbances can leave enduring imprints on microbial connectivity, potentially impairing ecosystem resilience (Wang S. et al., 2021; Yuan et al., 2021). In contrast, FL communities displayed the slowest recovery of network complexity, likely due to prolonged exposure to residual hydrological instability and nutrient pulses (Palmer and Ruhi, 2019; Shang et al., 2023).

The stage-specific shifts in microbial composition were exemplified by the contrasting dynamics of dominant taxa. During Inter_WSR3, *Gammaproteobacteria* (35.69% relative abundance in SS) and *Bacteroidia* (14.82% in SS) flourished in response to labile organic matter released from resuspended sediments (Song et al., 2012; Xia et al., 2018). Conversely, *Cyanobacteria* and *Anaerolineae* dominated FL and PA communities in Post-WSR, reflecting a shift toward photoautotrophic metabolisms as light availability and nutrient concentrations normalized (Xiao et al., 2017; Shi et al., 2020). These findings underscore the importance of linking microbial community structure to biogeochemical functions, such as organic matter degradation and nutrient cycling, which are tightly coupled to WSR-induced environmental gradients (Lu et al., 2022; Mu et al., 2024).

Collectively, these results challenge the notion that microbial communities in high-turbidity rivers rapidly rebound from disturbance. Instead, WSR induces a legacy of altered network topology and functional potential, with implications for long-term ecosystem stability. Addressing these impacts requires adaptive management strategies that account for stage-specific microbial vulnerabilities, particularly during sediment-release phases (Jia et al., 2023; Mu et al., 2024).

### Driving mechanisms of microbial community shifts: from environmental gradients to biogeochemical functions

The stage-specific shifts in microbial community structure were driven by dynamic physicochemical gradients imposed by WSR, which created distinct ecological niches for SS, PA, and FL microorganisms.

During the sediment-regulation stage (Inter_WSR3), extreme hydraulic turbulence and sediment resuspension led to peak concentrations of NO₃^−^ (3.10 mg/L), DIC (40.61 mg/L), and turbidity (77.80 NTU), alongside reduced DO (3.97 mg/L) (Supplementary Figure S1). These conditions favored copiotrophic taxa such as *Gammaproteobacteria* and *Bacteroidia*, which thrive in labile organic matter-rich environments. For instance, *Gammaproteobacteria* in SS reached 35.69% relative abundance during Inter_WSR3, reflecting their role in degrading sediment-released organic compounds (Song et al., 2012; Xia et al., 2018). Hierarchical partitioning further revealed that Chla (a proxy for phytoplankton biomass) dominated the SS community assembly during this phase, while NO₃^−^ and turbidity were key drivers for PA and FL communities (Figure 6).

In contrast, the pre-WSR stage (Pre_WSR) was characterized by stable hydrological conditions and lower nutrient fluxes, allowing *Nitrososphaeria* (AOA) and *Anaerolineae* to thrive in SS under anoxic conditions (Xiao et al., 2017; Wang et al., 2024).

The post-WSR recovery phase (Post_WSR) exhibited rebounding Chla levels (14.95 mg/L) and declining turbidity, shifting microbial drivers toward light availability and regenerated nutrients. For example, FL-*Cyanobacteria* and PA-*Anaerolineae* abundances increased by 15-fold and 3-fold, respectively, during Post_WSR, capitalizing on improved light penetration and residual nutrients (Figure 3C) (Xia et al., 2013).

PA communities exhibited unique responses to WSR-driven gradients. During Inter_WSR3, *Planctomycetes* and *Verrucomicrobia* dominated PA assemblages, likely due to their ability to break down complex organic matter within suspended particles (Crespo et al., 2013; Hu et al., 2022). These taxa showed positive correlations with turbidity and DIC (Figure 7D), suggesting their role in particle aggregation and inorganic carbon fixation under light-limited conditions. In contrast, FL communities relied more on dissolved substrates, with *Alphaproteobacteria* and *Bacteroidia* responding strongly to NO₃^−^ and TN fluctuations (Figure 7C).

The contrasting responses of the SS, PA, and FL compartments highlight their specialized ecological niches. For instance, SS microorganisms depend on sedimentary organic matter and nutrient stocks, while PA and FL communities are more dynamic, adjusting to hydrological and light-mediated resource availability (Palmer and Ruhi, 2019; Pan et al., 2022a, 2022b). This compartmentalization challenges the traditional view that FL communities dominate biogeochemical cycling in turbid rivers, emphasizing instead the critical role of particle-associated microbes in high-turbidity systems (Liu T. et al., 2018; Wang et al., 2021b).

Null model analysis revealed that underlying these stage-specific shifts in community structure was a fundamental change in ecological assembly processes. The extreme conditions experienced during Inter_WSR3 not only acted as a strong filter for specific copiotrophic lineages but also fundamentally reshaped the ecological forces governing the entire microbial community. In SS communities, the dominance of homogeneous selection during this stage provides a mechanistic explanation for the observed community patterns. The intense physical mixing effectively homogenized key environmental factors, such as nitrate and particulate matter, across the sediment habitat, creating a spatially uniform selective landscape. This powerful environmental filtering compelled microbial communities to converge, thereby overriding the influence of stochastic processes (Chen et al., 2022). Thus, our results collectively suggest that the extreme hydraulic disturbance enhanced deterministic assembly via habitat homogenization, rather than increasing the influence of stochastic processes.

Functionally, PICRUSt predictions of microbial gene content corroborated these structural shifts, demonstrating that the WSR process regulated microbial contributions to carbon, nitrogen, phosphorus, and sulfur cycling. For instance, the rTCA cycle remained stable in SS and PA but declined in FL, indicating that sediment-associated communities maintained their carbon fixation potential despite hydrological disturbance. This functional resilience in sediments was consistent with the abundance of dominant microbial taxa that rely on the rTCA pathway for carbon fixation, such as *Nitrososphaeria*, *Gammaproteobacteria,* and *Anaerolineae*, which were found to be abundant in SS (Garritano et al., 2022).

In the nitrogen cycle, while nitrate reduction dominated overall, its abundance decreased in PA and FL during Inter_WSR3, concurrently with an increase in denitrification. This shift reflects oxygen depletion and the subsequent transition toward anaerobic pathways, mirroring findings from river floodplain studies where hydrological fluctuations promote denitrification by creating anoxic microzones (Zhou et al., 2024). Similarly, the enrichment of comammox in SS highlights the resilience of sediment-based nitrifiers to hydraulic stress, a trend observed in other disturbed river systems (Pinto et al., 2015).

Phosphorus and sulfur cycles also responded significantly to WSR. Phosphorus transport dominated in SS and declined in PA at Inter_WSR3, consistent with increased phosphorus release under high turbidity and sediment resuspension (Withers and Jarvie, 2008). While sulfate reduction remained a dominant process across habitats, we observed a steady increase in sulfate oxidation in PA and FL during WSR. This suggests that turbulent mixing enhanced redox cycling and potentially microbial functional diversity in these compartments. Such shifts collectively support the notion that hydrodynamic regulation can accelerate coupled biogeochemical processes (Battin et al., 2016).

Overall, WSR disrupts biogeochemical coupling by altering environmental gradients, with legacy effects persisting in microbial network structure and functional redundancy. Addressing these impacts requires management strategies that account for stage-specific microbial vulnerabilities, particularly during sediment-release phases when network complexity is most disrupted (Wang et al., 2022; Mu et al., 2024).

### Microbial interactions and network complexity: implications for ecosystem functioning

The molecular ecological network analysis (MENs) revealed that water-sediment regulation (WSR) profoundly disrupts microbial interactions, with cascading effects on ecosystem functioning. During the sediment-regulation stage (Inter_WSR3), network complexity (nodes, links, modularity) peaked but failed to recover post-disturbance, indicating lasting destabilization of microbial communities. For example, the modularity of surface sediment (SS) networks dropped from 0.73 during Inter_WSR3 to 0.49 in Post-WSR, reflecting a collapse of modular structures critical for maintaining functional redundancy (Barberán et al., 2012). This loss of modularity suggests that WSR-induced disturbances weaken ecological resilience by disrupting synergistic microbial partnerships, such as mutualism and syntrophy, which underpin biogeochemical cycles (Xiao et al., 2017; Li et al., 2021).

Positive interactions (e.g., resource sharing, cross-feeding) among microbial taxa decreased significantly during Inter_WSR3, while negative interactions (e.g., competition) surged, particularly in particle-attached (PA) and free-living (FL) communities (Figure 5). For instance, PA-*Planctomycetes* and FL-*Alphaproteobacteria* exhibited stronger antagonistic relationships during WSR, possibly driven by competition for light and nutrients (Crespo et al., 2013; Yeh and Fuhrman, 2022). Such shifts in interaction patterns could impair nutrient cycling efficiency, as syntrophic partnerships (e.g., methanogenesis, denitrification) are disrupted (Wang S. et al., 2021; Yuan et al., 2021).

Stage-specific environmental drivers further shaped network topology. During Inter_WSR3, high turbidity and nutrient fluxes (e.g., NO₃^−^, DIC) favored generalist taxa with broad metabolic capabilities, leading to dense but less cohesive networks (Palmer and Ruhi, 2019; Shang et al., 2023). In contrast, post-WSR recovery was characterized by the re-emergence of specialist taxa (e.g., *Cyanobacteria* in FL), which formed tightly knit modules centered on light-driven processes (Xia et al., 2013; Pan et al., 2022a, 2022b). This dynamic highlights the trade-off between network stability and functional flexibility in response to disturbance.

Comparative analysis of low-turbidity systems (e.g., the Yangtze River) revealed stark contrasts in network resilience. For instance, FL communities in the Yellow River showed slower recovery of modularity compared to their clear-water counterparts, likely due to prolonged light limitation and sediment resuspension stress (Liu T. et al., 2018; Wang et al., 2021a). Moreover, the prominence of *Anaerolineae* and *Thermodesulfovibrionia* in SS networks during Inter_WSR3 underscored the importance of anaerobic guilds in high-turbidity systems, challenging assumptions derived from aerobic-dominated riverine ecosystems (Song et al., 2012; Xia et al., 2014).

These findings emphasize that WSR disrupts microbial co-occurrence patterns, with potential long-term consequences for ecosystem services. Strengthening network resilience through adaptive management—such as phased sediment release or habitat restoration—could mitigate ecological risks by preserving functional redundancy (Wang et al., 2022; Mu et al., 2024). Future studies integrating metagenomics and network dynamics are needed to unravel the mechanistic links between microbial interactions and biogeochemical processes in disturbed river systems.

### Contrasting patterns in high- vs. low-turbidity rivers: revisiting established paradigms

This study challenges the universality of paradigms established in low-turbidity river ecosystems by revealing distinct microbial responses in the high-turbidity Yellow River. In clear-water systems like the Yangtze River, chlorophyll a (Chla) and dissolved oxygen (DO) are widely recognized as primary drivers of bacterial community assembly (Green et al., 2012; Peiffer et al., 2021). However, our findings demonstrate that light limitation and sediment-water interactions override these drivers in turbid systems. For instance, Chla emerged as the dominant factor shaping surface sediment (SS) microbial communities during the sediment regulation stage (Inter_WSR3), whereas dissolved inorganic carbon (DIC) and turbidity governed particle-attached (PA) and free-living (FL) communities (Figure 6). This divergence reflects the unique niche partitioning in high-turbidity rivers, where suspended particles and sediment resuspension create microhabitats decoupled from surface water conditions (Xia et al., 2013; Pan et al., 2022a, 2022b).

Moreover, the prominence of taxa such as *Planctomycetes* and *Verrucomicrobia* in PA communities during Inter_WSR3 highlights functional adaptations specific to high-turbidity environments. These groups are traditionally associated with anoxic sediments in low-turbidity systems (Lage and Bondoso, 2014; Hu et al., 2022), yet in the Yellow River, they thrived in particle-associated niches driven by turbidity and DIC availability. Such observations contradict the assumption that particle-attached microbes universally rely on anoxic microenvironments, suggesting instead that physical transport and organic matter loading play pivotal roles in structuring their assemblages (Crespo et al., 2013; Yeh and Fuhrman, 2022).

The resilience of microbial networks also exhibited stark contrasts between turbidity regimes. While FL communities in low-turbidity rivers typically recover rapidly from disturbances (Palmer and Ruhi, 2019), those in the Yellow River displayed prolonged instability post-WSR. This delay was linked to residual hydrological perturbations and light limitation, which constrained the re-establishment of phototrophic taxa such as *Cyanobacteria* (Xiao et al., 2017; Shi et al., 2020). In contrast, SS communities rebounded more swiftly due to their reliance on physically stabilized sedimentary habitats, underscoring the buffering capacity of benthic environments in high-turbidity systems (Xia et al., 2013; Pan et al., 2022a, 2022b).

These findings demand a paradigm shift in how microbial ecology is studied in turbid rivers. Traditional frameworks emphasizing light and oxygen gradients must be revised to account for the complex interplay between sediment dynamics, particle-associated processes, and pulsed hydrological disturbances. For example, the unexpected dominance of *Anaerolineae* in SS during Inter_WSR3, despite oxic conditions, points to metabolic flexibility in response to episodic nutrient pulses rather than strict redox constraints (Song et al., 2012; Xia et al., 2014). Such insights highlight the limitations of extrapolating low-turbidity models to high-turbidity systems, where unique ecological rules govern microbial assembly and function.

Ultimately, this study underscores the need for context-specific management strategies in turbid rivers. Adaptive interventions—such as phased sediment release or artificial habitat enhancement—should prioritize the preservation of particle-associated microbial processes, which underpin critical biogeochemical functions such as organic matter degradation and nutrient cycling (Lu et al., 2022; Mu et al., 2024). By embracing the distinctiveness of high-turbidity ecosystems, researchers and practitioners can better navigate the trade-offs between sediment management goals and microbial ecological integrity.

### Management implications and future directions

The observed stage-specific microbial responses and network disruptions carry critical implications for optimizing water-sediment regulation (WSR) strategies in the Yellow River. Firstly, the pronounced destabilization of microbial networks during the sediment-regulation stage (Inter_WSR3) underscores the need to avoid pulsed sediment discharge events that coincide with peak hydraulic turbulence. Such disturbances not only disrupt nutrient cycling and carbon sequestration processes but also compromise ecosystem resilience by fragmenting microbial interactions. Implementing phased sediment release protocols—such as prolonging the duration of WSR or adopting variable discharge rates—could mitigate these risks by reducing the magnitude of environmental shocks to microbial communities.

Moreover, the delayed recovery of free-living (FL) microbial communities post-WSR highlights the importance of extending monitoring efforts beyond the immediate post-disturbance phases. FL microorganisms, which show the slowest rebound in network complexity, may serve as sentinel indicators of lingering ecological instability. Prioritizing the tracking of FL and particle-attached (PA) taxa—particularly during the transition from Inter_WSR3 to Post-WSR—could provide early warnings of functional shifts in biogeochemical cycles. For instance, declines in FL-*Alphaproteobacteria* or PA-*Planctomycetes* abundance might signal impaired nitrogen fixation or organic matter degradation capacities.

Enhancing habitat connectivity offers another promising avenue for restoring microbial network integrity. Artificial reefs, submerged vegetation, or sediment traps could help stabilize particle dynamics and promote recolonization by keystone taxa following WSR. Such interventions would complement existing efforts to manage light penetration and nutrient gradients, particularly in the sediment–water interface where *Gammaproteobacteria* and *Bacteroidia* thrive. Additionally, integrating microbial community metrics into ecological assessments could refine the evaluation of WSR outcomes, moving beyond traditional physicochemical parameters to encompass functional redundancy and network robustness.

Long-term monitoring is essential to assess whether legacy effects of repeated WSR cycles accumulate over time. Preliminary data suggest that repeated disturbances may lead to shifts in microbial community baselines, potentially favoring stress-tolerant taxa at the expense of biodiversity. Future studies should adopt a multi-year perspective to disentangle transient responses from sustained alterations, leveraging metagenomics and metabolomics to link community structure with functional gene expression. By adopting a holistic approach that balances sediment management goals with microbial ecological integrity, stakeholders can ensure the sustainability of the Yellow River ecosystem in the face of ongoing anthropogenic pressures.

## Conclusion

This study elucidates stage-specific microbial responses to water-sediment regulation (WSR) in the turbid Yellow River reservoir-river continuum. The sediment-regulation stage (Inter_WSR3) exerted the strongest disturbance due to pulsed hydraulic and biogeochemical gradients, marked by peak turbidity (77.80 NTU), NO₃^−^ (3.10 mg/L), and DIC (40.61 mg/L). These conditions favored copiotrophic taxa (*Gammaproteobacteria*, 35.69% in SS) while reconfiguring microbial networks, which exhibited peak complexity (nodes = 1,318; modularity = 0.73) but failed to recover Post_WSR. Legacy effects included reduced modularity and clustering coefficients, signaling weakened functional redundancy (such as in the phosphorus cycle, nitrogen cycle, and carbon cycle). Contrasting with low-turbidity systems, light limitation and sediment-water interactions dominated community assembly in the Yellow River. For example, *Anaerolineae* thrived in SS despite oxic conditions, reflecting metabolic flexibility to pulsed nutrients. Hierarchical partitioning identified Chla and turbidity as key drivers for SS and PA/FL communities, respectively, challenging paradigms that prioritize dissolved oxygen in clear-water rivers. To balance sediment management with ecological sustainability, we recommend implementing phased WSR to reduce network fragmentation, conducting targeted monitoring of FL/PA communities post-disturbance, and pursuing habitat restoration to enhance connectivity. Future studies should integrate metagenomics and long-term observations to elucidate the mechanistic links between microbial interactions and biogeochemical cycles in disturbed river systems. These findings deepen our understanding of high-turbidity river ecology and provide actionable insights for global river management.

## Data Availability

The datasets presented in this study can be found in online repositories. The names of the repository/repositories and accession number(s) can be found in the article/Supplementary material.

## Footnotes

^1^ http://ieg2.ou.edu/MENA/
