# Supplementary material for: Water-sediment regulation drives stage-specific microbial shifts and network complexity in the Yellow River reservoir-river continuum
Source: Front Microbiol. 2025 Oct 29;16:1640934. doi: 10.3389/fmicb.2025.1640934 (PMC12605214; doi:10.3389/fmicb.2025.1640934)
Supplement: Supplementary file 1 [file Data_Sheet_1.docx]

**Water-Sediment Regulation Drives Microbial Community Shifts in the Yellow River Reservoir-River Continuum**

**Yanmin Zhang^1^, Bo Zhao^1^, Zewei Gui^1^, Man Zhang^1^, Xulu Chang^1^, Guokun Yang^1^, Xiaolin Meng^1*^, Hongchen Jiang^2*^**

Running title: *WSR Alters Microbial Communities in Yellow River*

^1^College of Fisheries, Henan Normal University, Xinxiang 453007, PR China

^2^ School of Life Sciences, Henan University, Kaifeng 475001, China

*****Correspondence:

Hongchen Jiang, E-mail: [jiangh@henu.edu.cn](mailto:jiangh@henu.edu.cn)

Xiaolin Meng, E-mail: [mengxiaolinqdio@126.com](mailto:mengxiaolinqdio@126.com)

**Table S1** The alpha diversity indices of SS, PA and FL in different stages of WSR( mean values from 8 sampling sites, with three replicates for each of PA, FL, and SS at each stage, n = 24).

|  | **Stages of WSR** | **Richness** | **Shannon** | **Simpson** | **Pielou** | **Good’s_**  **coverage** |
| --- | --- | --- | --- | --- | --- | --- |
| **SS** | Pre_WSR | 2175.75 | 9.41 | 0.9911 | 0.8494 | 1 |
|  | Inter_WSR1 | 2046.63 | 9.62 | 0.9956 | 0.8756 | 1 |
|  | Inter_WSR2 | 2093.00 | 9.80 | 0.9971 | 0.8901 | 1 |
|  | Inter_WSR3 | 2616.13 | 9.23 | 0.9475 | 0.8130 | 1 |
|  | Post_WSR | 2884.75 | 10.17 | 0.9965 | 0.8881 | 1 |
|  | **Average** | **2326.25^a^** | **9.65 ^a^** | **0.9856** | **0.8632 ^a^** | **1** |
| **PA** | Pre_WSR | 729.88 | 6.69 | 0.9544 | 0.7034 | 1 |
|  | Inter_WSR1 | 633.38 | 6.17 | 0.9424 | 0.6631 | 1 |
|  | Inter_WSR2 | 640.88 | 6.41 | 0.9484 | 0.6880 | 1 |
|  | Inter_WSR3 | 1009.63 | 7.17 | 0.9709 | 0.7205 | 1 |
|  | Post_WSR | 801.38 | 6.88 | 0.9659 | 0.7161 | 1 |
|  | **Average** | **763.03^b^** | **6.66 ^b^** | **0.9564** | **0.6982 ^b^** |  |
| **FL** | Pre_WSR | 491.13 | 6.13 | 0.9541 | 0.6878 | 1 |
|  | Inter_WSR1 | 430.25 | 5.87 | 0.9287 | 0.6713 | 1 |
|  | Inter_WSR2 | 493.88 | 6.30 | 0.9582 | 0.7055 | 1 |
|  | Inter_WSR3 | 725.13 | 6.97 | 0.9739 | 0.7371 | 1 |
|  | Post_WSR | 560.50 | 6.69 | 0.9714 | 0.7339 | 1 |
|  | **Average** | **540.18^bc^** | **6.39 ^bc^** | **0.9573** | **0.7071^bc^** |  |

Note: The average indices of SS, PA and FL with different superscripts are significantly different (*P* < 0.05).

**Table S2** Abundant phyla (or class) (relative abundance≥0.5%) of SS in different stages of WSR (mean values from 8 sampling sites, with three replicates for each of PA, FL, and SS at each stage, n = 24).

| **Phyla(or class)** | **Relative abundance (%)** | | | | | **Average** |
| --- | --- | --- | --- | --- | --- | --- |
|  | **Pre_WSR** | **Inter_WSR1** | **Inter_WSR2** | **Inter_WSR3** | **Post_WSR** | **(%)** |
| *Gammaproteobacteria* | 18.37 | 17.90 | 26.34 | 35.69 | 26.31 | 24.92 |
| *Alphaproteobacteria* | 6.99 | 4.95 | 4.44 | 3.66 | 3.17 | 4.64 |
| *Actinobacteria* | 2.96 | 3.41 | 2.44 | 1.11 | 0.98 | 2.18 |
| *Bacteroidia* | 14.97 | 15.20 | 16.72 | 9.72 | 14.82 | 14.29 |
| *Planctomycetes* | 2.81 | 2.40 | 2.37 | 1.88 | 3.82 | 2.66 |
| *Cyanobacteriia* | 8.54 | 6.04 | 0.65 | 0.27 | 3.28 | 3.76 |
| *Acidimicrobiia* | 0.31 | 0.42 | 0.37 | 0.50 | 0.53 | 0.43 |
| *Verrucomicrobiae* | 3.62 | 2.55 | 2.85 | 2.04 | 3.36 | 2.88 |
| *Nitrososphaeria* | 9.62 | 9.43 | 8.35 | 4.42 | 4.01 | 7.17 |
| *Anaerolineae* | 5.37 | 5.67 | 4.49 | 6.30 | 7.26 | 5.82 |
| *Thermodesulfovibrionia* | 2.40 | 3.23 | 2.08 | 3.74 | 3.63 | 3.02 |
| Others | 24.03 | 28.82 | 28.90 | 30.64 | 28.81 | 28.24 |

**Table S3** Abundant phyla (or class) (relative abundance≥0.5%) of PA in different stages of WSR (mean values from 8 sampling sites, with three replicates for each of PA, FL, and SS at each stage, n = 24).

| **Phyla(or class)** | **Relative abundance (%)** | | | | | **Average（%）** |
| --- | --- | --- | --- | --- | --- | --- |
|  | **Pre_WSR** | **Inter_WSR1** | **Inter_WSR2** | **Inter_WSR3** | **Post_WSR** |  |
| *Gammaproteobacteria* | 23.51 | 28.67 | 22.47 | 22.83 | 20.80 | 23.66 |
| *Alphaproteobacteria* | 14.88 | 14.01 | 19.52 | 16.44 | 14.41 | 15.85 |
| *Actinobacteria* | 9.30 | 9.81 | 10.69 | 10.21 | 11.21 | 10.24 |
| *Bacteroidia* | 15.19 | 14.23 | 7.65 | 9.74 | 8.31 | 11.02 |
| *Planctomycetes* | 14.18 | 11.20 | 11.12 | 9.94 | 6.66 | 10.62 |
| *Cyanobacteriia* | 1.23 | 7.31 | 8.30 | 5.74 | 20.27 | 8.57 |
| *Acidimicrobiia* | 5.40 | 4.62 | 10.64 | 9.92 | 6.02 | 7.32 |
| *Verrucomicrobiae* | 9.49 | 5.64 | 2.88 | 2.30 | 2.18 | 4.50 |
| *Nitrososphaeria* | 0.01 | 0.01 | 0.01 | 0.26 | 0.01 | 0.06 |
| *Anaerolineae* | 0.03 | 0.04 | 0.03 | 0.20 | 0.23 | 0.11 |
| *Thermodesulfovibrionia* | 0.01 | 0.00 | 0.01 | 0.11 | 0.01 | 0.03 |
| Others | 6.77 | 4.46 | 6.69 | 12.31 | 9.90 | 8.03 |

**Table S4** Abundant phyla (or class) (relative abundance≥0.5%) of FL in different stages of WSR (mean values from 8 sampling sites, with three replicates for each of PA, FL, and SS at each stage, n = 24).

| **Phyla(or class)** | **Relative abundance (%)** | | | | | **Average** |
| --- | --- | --- | --- | --- | --- | --- |
|  | **Pre_WSR** | **Inter_WSR1** | **Inter_WSR2** | **Inter_WSR3** | **Post_WSR** | **(%)** |
| *Gammaproteobacteria* | 19.52 | 28.07 | 20.23 | 31.65 | 19.22 | 23.74 |
| *Alphaproteobacteria* | 20.33 | 25.85 | 20.21 | 23.80 | 11.22 | 20.28 |
| *Actinobacteria* | 29.42 | 21.34 | 20.38 | 15.84 | 23.30 | 22.06 |
| *Bacteroidia* | 9.87 | 10.97 | 4.01 | 5.36 | 6.15 | 7.27 |
| *Planctomycetes* | 5.38 | 3.68 | 9.77 | 4.43 | 4.30 | 5.51 |
| *Cyanobacteriia* | 0.13 | 0.73 | 4.23 | 3.56 | 15.91 | 4.91 |
| *Acidimicrobiia* | 7.25 | 4.41 | 14.47 | 6.76 | 12.54 | 9.09 |
| *Verrucomicrobiae* | 5.37 | 2.97 | 3.11 | 0.77 | 2.21 | 2.89 |
| *Nitrososphaeria* | 0.00 | 0.00 | 0.00 | 0.14 | 0.01 | 0.03 |
| *Anaerolineae* | 0.01 | 0.00 | 0.01 | 0.03 | 0.11 | 0.03 |
| *Thermodesulfovibrionia* | 0.00 | 0.00 | 0.00 | 0.02 | 0.00 | 0.00 |
| Others | 2.73 | 1.96 | 3.57 | 7.65 | 5.02 | 4.19 |

**Table S5** Hierarchical partitioning (HP) analysis shows the relative contributions of physicochemical factors to the SS-, PA-, and FL-related microbial community structures. *, *P* < 0.05.

|  |  | **Relative contribution** | | | | | | |
| --- | --- | --- | --- | --- | --- | --- | --- | --- |
| **Microbial community** | **Stages of WSR** | **Turbidity** | **Chla** | **NH_4_^+^** | **NO_3_^-^** | **TN** | **TP** | **DIC** |
| **SS** | **Pre_WSR** | 0.10 | 0.20 | 0.11 | 0.09 | 0.18 | 0.14 | 0.18 |
|  | **Inter_WSR1** | 0.21 | 0.05 | 0.10 | 0.25 | 0.09 | 0.08 | 0.21 |
|  | **Inter_WSR2** | 0.10 | 0.22 | 0.19 | 0.26* | 0.13 | 0.06 | 0.05 |
|  | **Inter_WSR3** | 0.07 | 0.30* | 0.08 | 0.19 | 0.10 | 0.20 | 0.07 |
|  | **Post_WSR** | 0.08 | 0.33* | 0.14 | 0.03 | 0.11 | 0.11 | 0.21 |
| **PA** | **Pre_WSR** | 0.10 | 0.18 | 0.16 | 0.09 | 0.13 | 0.13 | 0.20 |
|  | **Inter_WSR1** | 0.13 | 0.04 | 0.11 | 0.20 | 0.16 | 0.16 | 0.19 |
|  | **Inter_WSR2** | 0.08 | 0.18 | 0.17 | 0.28* | 0.11 | 0.10 | 0.06 |
|  | **Inter_WSR3** | 0.25 | 0.16 | 0.03 | 0.10 | 0.10 | 0.13 | 0.23 |
|  | **Post_WSR** | 0.19 | 0.13 | 0.23 | 0.06 | 0.15 | 0.08 | 0.16 |
| **FL** | **Pre_WSR** | 0.11 | 0.17 | 0.09 | 0.11 | 0.17 | 0.12 | 0.22 |
|  | **Inter_WSR1** | 0.17 | 0.08 | 0.12 | 0.20 | 0.14 | 0.11 | 0.19 |
|  | **Inter_WSR2** | 0.12 | 0.11 | 0.10 | 0.29* | 0.15 | 0.15 | 0.09 |
|  | **Inter_WSR3** | 0.19 | 0.17 | 0.10 | 0.10 | 0.09 | 0.18 | 0.16 |
|  | **Post_WSR** | 0.25* | 0.07 | 0.17 | 0.10 | 0.18 | 0.11 | 0.12 |

**Table S6** Two-way PERMANOVA results testing the effects of WSR stage and sampling site on the community structure of different microbial types.

|  | **Source** | **Df** | **SumOfSqs** | **F** | **R^2^(%)** | ***P*_value** |
| --- | --- | --- | --- | --- | --- | --- |
| **SS** | Stage | 4 | 2.44 | 6.17 | 38.46 | 0.001 |
|  | Site | 7 | 1.14 | 1.65 | 17.94 | 0.001 |
|  | Residual | 28 | 2.76 |  | 43.60 |  |
| **PA** | Stage | 4 | 1.57 | 1.86 | 15.86 | 0.001 |
|  | Site | 7 | 2.42 | 1.64 | 24.49 | 0.001 |
|  | Residual | 28 | 5.90 |  | 59.65 |  |
| **FL** | Stage | 4 | 2.37 | 3.91 | 24.54 | 0.001 |
|  | Site | 7 | 3.05 | 2.87 | 31.53 | 0.003 |
|  | Residual | 28 | 4.24 |  | 43.92 |  |

**Table S7** Pearson correlations between network properties, sequencing depth, and community diversity.

|  | **Average Sequencing Depth** | **Shannon Diversity Index** |
| --- | --- | --- |
| **Edges** | 0.130 | **0.766**** |
| **Modularity** | -0.069 | -0.232 |
| **Clustering Coefficient** | 0.169 | -0.022 |

Note: Values represent Pearson correlation coefficients. ** indicates *P* < 0.01 (two-tailed).

**Fig.** **S1** Physicochemical variations among the five stages of WSR. Significance was tested using one-way ANOVA, different superscript letters indicated significant difference (*P*< 0.05), whereas identical or unmarked letters indicate no significant differences (*P* > 0.05).


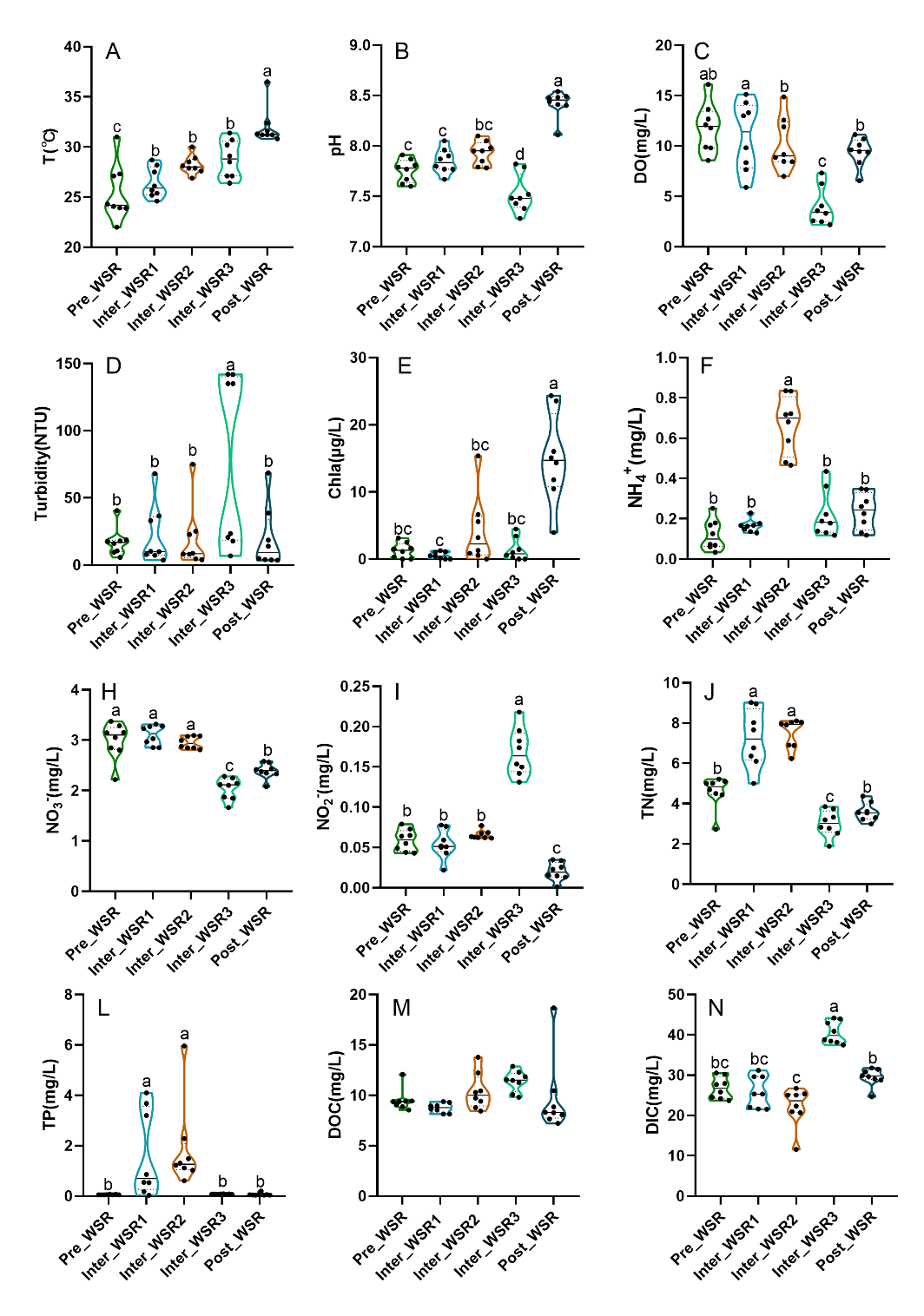


**Fig. S2 Relative abundance shifts of carbon cycle pathway across WSR stages (Predict by PICRUSt2 from 16S rRNA gene high-throughput sequencing data). A.** CBB cycle, Calvin-Benson-Bassham cycle; **B.** rTCA cycle, Reductive tricarboxylic acid cycle; **C.** 3HP cycle, 3-hydroxypropionic acid cycle; **D.** 3HP/4HB cycle, 3-hydroxypropionic acid/4-hydroxybutanoic acid cycle; **E.** DC/4HB cycle, Dicarboxylic Acid/4-Hydroxybutyrate cycle; **F.** WL pathway, Wood-Ljungdahl pathway. **G.** Methanogenesis. **H.** Methane oxidation. Significance was determined by one-way ANOVA: “∗∗∗”, *P* < 0.001; “∗∗”, *P* < 0.01; and “∗”, *P* < 0.05. Data represent mean values from 8 sampling sites, with three replicates each for PA, FL, and SS per stage (n = 24).

**Fig. S3 Relative abundance shifts of nitrogen cycle pathway across WSR stages (Predict by PICRUSt2 from 16S rRNA gene high-throughput sequencing data). A.** Nitrogen fixation. **B.** Nitrate reduction. **C.** Denitrification. **D.** Nitrification. **E.** Comammox. **F.** Anammox. Significance was determined by one-way ANOVA: “∗∗∗”, *P* < 0.001; “∗∗”, *P* < 0.01; and “∗”, *P* < 0.05. Data represent mean values from 8 sampling sites, with three replicates each for PA, FL, and SS per stage (n = 24).

**Fig. S4 Relative abundance shifts of phosphoyus and sulfur cycle pathway across WSR stages (Predict by PICRUSt2 from 16S rRNA gene high-throughput sequencing data). A.** Organic P mineralization. **B.** P transportion. **C.** Inorganic P solubilization. **D.** P regulation. **E.** Sulfate reduction. **F.** Sulfur oxidation. Significance was determined by one-way ANOVA: “∗∗∗”, *P* < 0.001; “∗∗”, *P* < 0.01; and “∗”, *P* < 0.05. Data represent mean values from 8 sampling sites, with three replicates each for PA, FL, and SS per stage (n = 24).

**Fig. S5 Null model analysis of microbial community assembly processes across WSR stages. A, B.** SS communities. **C, D.** PA communities. **E, F.** FL communities. **A, C, E.** Pairwise comparison results of the βNTI index. The dashed lines at βNTI = +2 and -2 delineate the thresholds for deterministic processes. **B, D, F.** Quantitative partitioning of the relative importance of ecological processes.

**
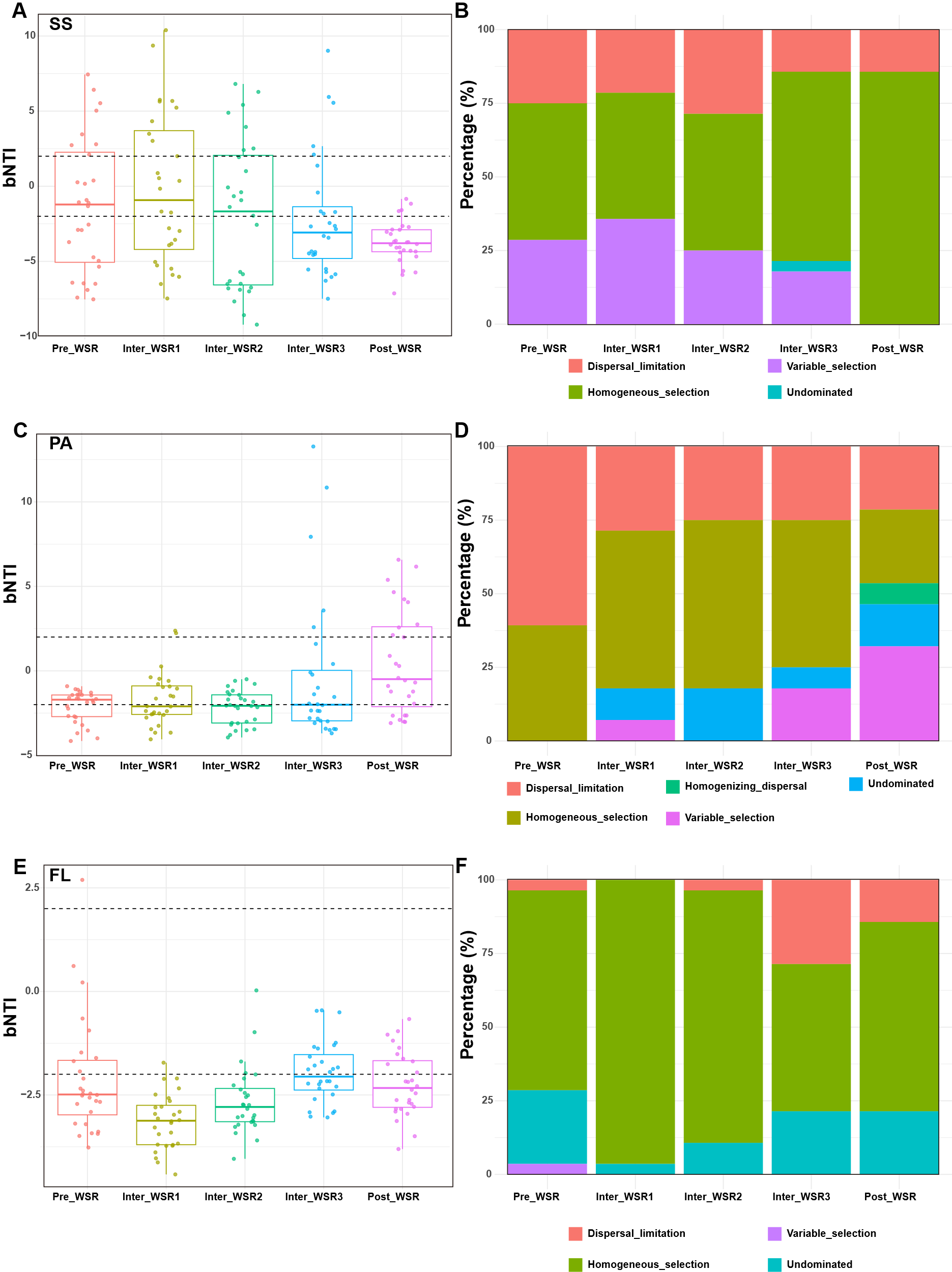
**
